# Metal Ion–Induced Cross‐Linking in Mucin‐Inspired Peptide Hydrogels

**DOI:** 10.1002/psc.70059

**Published:** 2025-09-23

**Authors:** Annelie Puhlmann, Cihan Baydaroglu, Boris Schade, Michael Gradzielski, Beate Koksch

**Affiliations:** ^1^ Institute of Chemistry and Biochemistry Freie Universität Berlin Berlin Germany; ^2^ Stranski‐Laboratorium für Physikalische und Theoretische Chemie, Institut für Chemie Technische Universität Berlin Berlin Germany; ^3^ Research Center of Electron Microscopy Freie Universität Berlin Berlin Germany

**Keywords:** cross‐linking, glycopeptide, histidine, metal coordination, peptide hydrogels

## Abstract

Mucus is the biological hydrogel that lines the mucosal surfaces of mammals and acts as a protective barrier. Its main proteinaceous component is mucin, the high molecular weight, degree of glycosylation, and hardly uniquely defined nature of which hamper precise structures/property investigations based on biological samples. In contrast, chemically precisely defined peptide model systems inspired by such natural glycoproteins represent synthetically readily obtainable tools with excellent properties for both fundamental research and biomedical applications. Herein, we report the design and characterization of a library of histidine‐ and monosaccharide‐containing coiled coil peptides that form hydrogels to different degrees in the presence of divalent metal ions Cu^2+^, Zn^2+^, Ca^2+^, and Fe^2+^. Using rheology, circular dichroism, and transmission electron microscopy, we determined the viscoelastic properties and global structures of these glycopeptide materials. This study reflects the interplay between glycan identity, histidine position, and divalent metal ion on the mechanical strength of these hydrogels.

## Graphical abstract

Short fiber‐forming peptides were modified by histidine substitution and mannose or galactose attachment and combined with different metal ions. Although no generalizable trends were observed, Lys to His substitution at position 25 with galactose in the presence of copper produced a species with highly favorable viscoelastic properties.

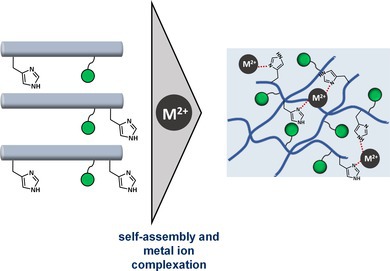

## 1 Introduction

Hydrogels are three‐dimensional polymer networks that retain large quantities of water. Their tunability and functionality make them suitable not only in natural systems but also in many biomedical and bioengineering applications ranging from drug‐delivery systems to sensors as well as everyday objects such as soft contact lenses [1, 2, 3, 4].

These networks of synthetic or biological origin are held together by different modes of cross‐linking, which can be influenced by external stimuli, for example, heat, cations, or pH [5]. The changes in their properties that result from stimulus exposure are of fundamental importance for understanding their functions [6].

Mucus is a biological hydrogel that lines the mucosal surfaces in the human body, such as those in the stomach, eyes, and lungs [7, 8, 9, 10]. It acts as a barrier that protects cells from physical stress and transports nutrients across the cell membrane. Mucins are the building blocks of mucus and consist of glycoprotein‐rich networks that act as a kind of storage receptacle for different glycans. These networks are responsible for cell adhesion and signaling. Mucins are characterized by domains rich in proline, threonine, and serine (PTS) or cysteine‐rich regions and differ in their precise composition of glycans and peptides. Because of this variety in composition, mucins are complex structures, and their assembly by means of chemical peptide and glycan synthesis is challenging. Nevertheless, the study of structure–activity relationships of mucins is essential to further understand their precise biological functions in the human body in health and disease [11].

This need led to the synthesis of multiple mucin‐inspired peptide systems in recent years [12, 13]. In particular, the coiled coil (CC) secondary structure motif has been used for the production of self‐assembling bioactive materials [14, 15, 16, 17]. In more recent studies, CC‐scaffold hFF03 (hFF: hydrogelating fiber‐forming peptide scaffold) functionalized with cell adhesion motifs and mannose has been described by Koksch et al. [18, 19] This motif was further developed for a systematic investigation of the impact of glycan functionalization on hydrogel properties that were studied by oscillatory shear rheology [20].

As mentioned above, hydrogels typically display various types of cross linkages, not the least of which can originate from trace elements in biological systems [21]. The influence of trace elements on mucus has been clearly shown for diseases such as cystic fibrosis or chronic hypertrophic rhinosinusitis, in which increases in metal ion concentration are observed in the disease state [22, 23].

These cross‐linking interactions between trace elements and peptides arise through potential coordination sites presented by amino acid side chains, such as methionine or histidine. For example, histidine‐metal coordination is used by nature to enhance the mechanical properties of hydrogels, such as hardness, toughness, or adhesion [24, 25]. In the context of this work, we chose histidine as a coordination site, as it offers two pH‐switchable cross‐linking acceptors as different pKa values (6.5 and 14) lead to deprotonation of either the τ‐ or π‐nitrogen within the imidazole ring, which in turn directs coordination [26].

Herein, histidine was incorporated at the f‐position of the fiber‐forming CC motif, which is accessible to divalent metal ions. The combination of the peptide with two monosaccharide building blocks (mannose and galactose) led to the synthesis of nine different (glyco)peptides, which were analyzed for their structure (circular dichroism [CD] and transmission electron microscopy) and viscoelastic properties (rheology) under physiological conditions, both in the absence and presence of four divalent metal ions (Cu^2+^, Zn^2+^, Ca^2+^, and Fe^2+^), which have been reported to be components trace elements of natural mucins [27, 28, 29, 30].

## 2 Materials and Methods

### 2.1 Synthesis of Peptides

All peptide syntheses were conducted according to solid‐phase peptide synthesis (SPPS) using the Fmoc strategy by means of a Liberty Blue (CEM Corporation, Matthews, NC, USA) microwave‐assisted peptide synthesizer (details given in the Supporting Information). Using Oxyma/1,3‐diisopropylcarbodiimide (DIC) as an activating agent, syntheses were performed at the 0.1 mmol scale. Peptides were synthesized on preloaded Fmoc‐Leu‐Wang resin (0.24 mmol/g) from Novabiochem. 1‐Amino‐1‐deoxy‐β‐D‐mannopyrannose and 1‐amino‐1‐deoxy‐β‐D‐galactose (Santa Cruz Biotechnology, USA) were coupled via glutaric anhydride linker to the glycan‐peptide conjugates on the solid phase. After full cleavage and HPLC purification, the anions were exchanged from trifluoroacetic acid to chloride (details given in the Supporting Information).

### 2.2 Glycan Functionalization of Peptides

The synthesis of the glycan peptide conjugates can be divided into two parts. First, the peptides were synthesized using SPPS. Second, whereas the sequence was still on resin, an orthogonal protecting group on lysine residue 18 was selectively deprotected, and the glycan was attached to this position via glutaric anhydride linker.

After full‐length synthesis, the Mmt protecting group of lysine 18 was cleaved using a mixture of dichloromethane (DCM), trifluoroethanol (TFE), and acetic acid (AcOH) (7:2:1, v/v; 1× 30 min, 1× 5 h). Afterwards, the free amino function was treated with a solution of glutaric anhydride (10 equiv.) and DIPEA (catalytic) in DMF. Lastly, the resulting carboxy function of the linker was activated with a solution of COMU (2 equiv.) and DIPEA (4 equiv.) in DMF for 1 min in an ultrasonic bath at 40°C. Afterwards, a solution of the respective glycan (2 equiv.) in DMF was added. Full cleavage of the peptides was achieved by treating the resin with a mixture of TFA, dichloromethane, and TIPS (50:45:5 v/v) for 45 min. After filtration, volatiles were evaporated, and the peptides precipitated in cold diethyl ether, centrifuged, dissolved in water, and lyophilized. Purification was performed using preparative RP‐HPLC (for details, see the Supporting Information).

### 2.3 Peptide Initial Characterization

Tables S1–S12 contain all initial physical characterizations of the synthesized peptide library.

### 2.4 Sample Preparation

Purified peptides were dissolved in 1,1,1,3,3,3‐hexafluoroisopropanol (HFIP) or water, depending on solubility, at roughly 1 mg per 100 μL and sonicated for 10 min. Peptide stock solution (10 μL) was evaporated in a gentle stream of nitrogen gas, and the resulting peptide film was dissolved in 1 mL of Dulbecco's phosphate‐buffered saline (DPBS) containing 6 M guanidine hydrochloride. Absorbance of this solution at 320 nm was measured with a Varian Cary 50 photometer (Varian Medical Systems, Palo Alto, CA, USA). The calibration curve of dipeptide H‐2‐Abz‐Gly‐OH was used to calculate the concentration of the stock solution (for details, see the Supporting Information).

Appropriate amounts of the HFIP peptide stock solutions of known concentration were then evaporated under a gentle stream of nitrogen, and the resulting peptide residue was dissolved as described in the respective experimental section below.

### 2.5 Metal Coordination

Metal stock solutions (1 M) were prepared using copper(II) chloride dihydrate (Thermo Fisher scientific, Acros Organics), zinc(II) chloride (Merck), iron(II) chloride tetrahydrate (Sigma‐Aldrich) or calcium(II) chloride dihydrate (Fluka Biochemika) dissolved in 1 mL water (pH 7.4); pH was adjusted where needed with diluted NaOH (1 M) and HCl (1 M).

Peptide stock solutions (1.65 mM) were evaporated, and the residue redissolved in water. The pH was adjusted to 7.4 (where needed with diluted NaOH or HCl), and the metal stock solutions were added. Water (pH 7.4) was added to reach a total volume of 150 μL (rheology) or 50 μL (CD). Peptides were incubated over 20 h at 37°C to reach equilibration in an Eppendorf ThermoMixer F (Eppendorf, Hamburg, Germany). The samples were centrifuged, vortexed for 10 s, and centrifuged again.

### 2.6 CD Spectroscopy

CD spectra were recorded using a Jasco J‐810 spectropolarimeter. Temperature was regulated using a Jasco PTC‐432S Peltier temperature control system, which was connected to the spectropolarimeter. Measurements were performed at 37°C. CD spectra of hydrogel samples were recorded using demountable Quartz Suprasil cuvettes (Hellma Analytics, Mühlheim, Germany) with a path length of 0.1 mm. Spectra were background‐corrected by subtraction of water pH 7.4 spectra at 37°C and normalized by path length, molar concentration, and number of amide bonds (Equation (1)).

(1) $$\left[\theta \right]=\frac{\theta_{\mathrm{obs}}}{c*n*l*10000}10^{3}\deg\;\mathrm{dmo}l^{-1}{\mathrm{cm}}^{2}\text{residu}e^{-1}$$

$$\begin{array}{c} \theta_{\mathrm{obs}}=\text{ellipticity measured},c=\text{concentration}\;\left[M\right],\\ \;n=\text{number of amide bonds},l=\text{path length}\;\left[\mathrm{cm}\right] \end{array}$$

Samples were prepared by dissolving 0.5 wt% (also 0.1 or 0.25 wt% for free samples at initial characterization as shown in Tables S1–S12) peptides in water (30 μL). The pH was adjusted to 7.4 by the addition of 1 M aqueous NaOH or HCl. The metal‐stock solution was added, and the volume was adjusted to a total volume of 50 μL with water at pH 7.4. The samples were vortexed and incubated for 20 h at 37°C.

### 2.7 Rheology

Rheology measurements were performed on an Anton Paar MCR 502 WESP temperature‐controlled rheometer in strain‐imposed mode at 37°C. A chromium oxide–coated cone‐plate measurement system was used with a diameter of 25 mm, a cone truncation (gap width) of 48 μm, and a cone angle of 2°. Temperature control was performed using a Peltier measuring system combined with a Peltier hood to ensure a minimized temperature gradient throughout the sample.

Samples were prepared by dissolving 0.5 wt% peptides in water (100 μL) by means of oscillatory shear rheology. The pH was adjusted to 7.4 by the addition of 1 M aqueous NaOH or HCl. The appropriate metal‐stock solution was added, and the volume was adjusted to a total volume of 150 μL with water pH 7.4. The samples were vortexed and incubated for 20 h at 37°C.

### 2.8 Transmission Electron Microscopy

Cryo‐transmission electron microscopy (Cryo‐TEM) was performed using 0.1 mM peptide hydrogel samples. 4 μL aliquots were applied to perforated carbon film covered microscopical grids (200 mesh, R1/4 batch of Quantifoil, MicroTools GmbH, Jena, Germany). Grids were cleaned with chloroform and hydrophilized by 60 s glow discharging at 8 W in BALTEC MED 020 device (Leica Microsystems, Wetzlar, Germany) before use. The samples were then plunge frozen in a Vitrobot Mark IV (Thermo Fisher Scientific Inc., Waltham, Massachusetts, USA) using liquid ethane as cryogen. The samples were then transferred to a Talos Arctica electron microscope equipped with a high‐brightness field‐emission gun (XFEG) operated at 200 kV acceleration voltage. Micrographs were acquired on a Falcon 3 direct electron detector (Thermo Fisher Scientific Inc., Waltham, Massachusetts, USA) at nominal magnifications of 28,000×.

## 3 Results and Discussion

### 3.1 Design

The 26‐residue peptide hFF03 forms an α‐helical coiled‐coil dimer with a length of 4 nm [31]. Proksch et al. modified the hFF03 model with different glycans at lysine position 18 and analyzed them for their viscoelastic properties and structure [20].

**FIGURE 1:**
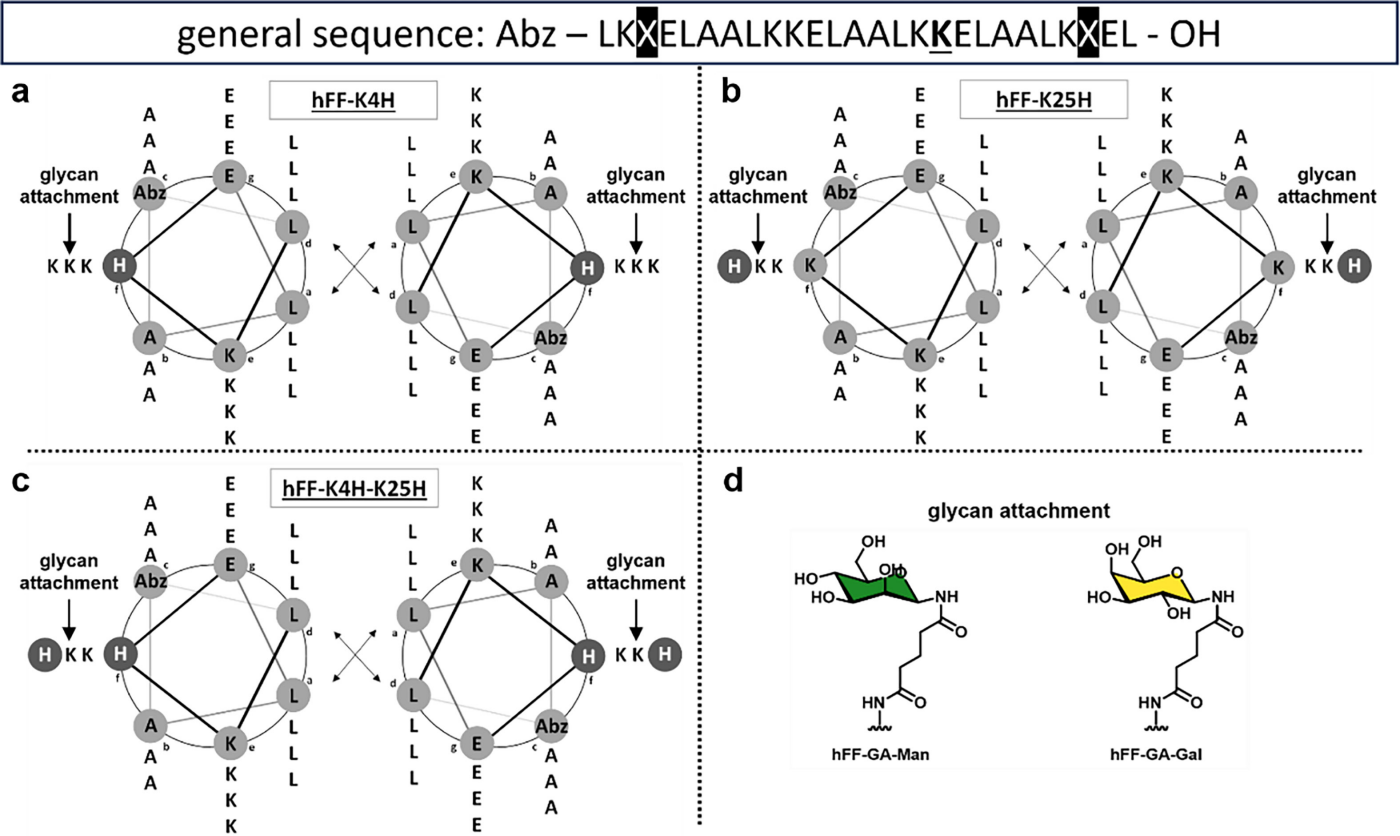
(a–c) Helical wheel diagram of peptide primary structures in which X represents either K or H and the central underlined K indicates the site of monosaccharide modification via glutaric anhydride linker GA. (d) Chemical structure of respective liner monosaccharides.

The chromophore 2‐Abz‐OH is present at position 1 as a tool for concentration determination. Histidine was incorporated at the 4th and/or 25th position (f‐position of CC) of the peptide, allowing sufficient accessibility for the divalent metal ions. This resulted in the synthesis of peptides hFF‐K4H, hFF‐K25H, and hFF‐K4H‐K25H (Figure 1). To gain insight into the role of glycans in mucin‐inspired peptides, the two commercially available 1‐aminoglycans mannose and galactose were used for the synthesis using glutaric anhydride (GA) to convert side‐chain functionalization of lysine. Thus, peptides hFF‐K4H, hFF‐K25H, and hFF‐K4H‐K25H were synthesized along with their corresponding linker‐glycan conjugates shown in Figure 1 and listed in Table 1.

**TABLE 1:** Peptide library containing all studied hFF variants; peptide marked with “*” represent published sequence that serve here as control [19].

Abbreviation	Position 1	Position 2 (left)–27 (right)
*hFF	Abz	LKKELAALKKELAALKKELAALKKEL
hFF‐GA‐Man	Abz	LKKELAALKKELAALK**K**_(GA‐Man)_ELAALKKEL
hFF‐GA‐Gal	Abz	LKKELAALKKELAALK**K**_(GA‐Gal)_ELAALKKEL
hFF‐K4H	Abz	LK **H** ELAALKKELAALKKELAALKKEL
hFF‐K4H‐GA‐Man	Abz	LK **H** ELAALKKELAALK**K**_(GA‐Man)_ELAALKKEL
hFF‐K4H‐GA‐Gal	Abz	LK **H** ELAALKKELAALK**K**_(GA‐Gal)_ELAALKKEL
hFF‐K25H	Abz	LKKELAALKKELAALKKELAALK **H** EL
hFF‐K25H‐GA‐Man	Abz	LKKELAALKKELAALK**K**_(GA‐Man)_ELAALK **H** EL
hFF‐K25H‐GA‐Gal	Abz	LKKELAALKKELAALK**K**_(GA‐Gal)_ELAALK **H** EL
hFF‐K4H‐K25H	Abz	LK **H** ELAALKKAAEKLKKELAALK **H** EL
hFF‐K4H‐K25H‐GA‐Man	Abz	LK **H** ELAALKKELAALK**K**_(GA‐Man)_ELAALK **H** EL
hFF‐K4H‐K25H‐GA‐Gal	Abz	LK **H** ELAALKKELAALK**K**_(GA‐Gal)_ELAALK **H** EL

### 3.2 CD Spectroscopy

**FIGURE 2:**
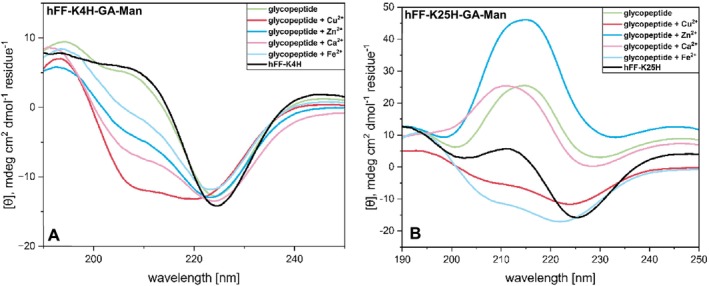
CD spectra of histidine‐monosubstituted mannose‐functionalized peptides at 0.5 wt% with and without metal cations in water pH 7.4 at 37°C, with CD spectra (black) of non‐glycosylated peptide.

**FIGURE 3:**
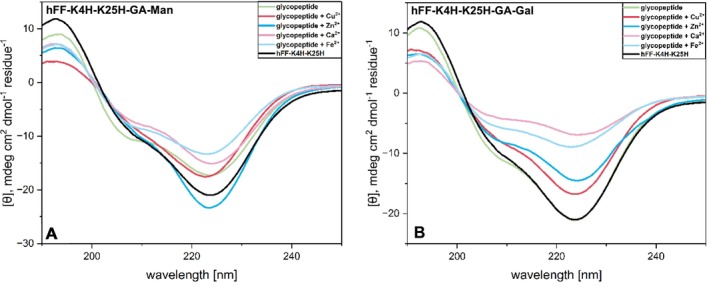
CD spectra of histidine‐disubstituted mannose‐ and galactose‐ functionalized peptide at 0.5 wt% on addition of different metal cations in water at pH 7.0 and 37°C.

The overall structure of all peptide variants was analyzed by means of CD spectroscopy at concentrations of 0.1, 0.25, and 0.5 wt% (Tables S1–S12). The impact of different divalent metal ions on this global structure, however, was only investigated at the highest peptide concentration of 0.5 wt% (Figures 2 and 3 and Figures S1–S8). Proksch et al. had shown in a prior study that peptides of the hFF series form higher ordered structures at a concentration of 0.5 wt%; this was determined by a decrease in the intensity ratio between the minimum at 208 and that at 222 nm, which for an ideal alpha‐helix is essentially 1:1 [20]. As mentioned above, selection of the metal cations was based on their importance in the context of mucus [27, 28, 29, 30]. Samples were prepared in pure water because the metal salts precipitate in phosphate buffers and the peptides precipitate in organic buffers. All experiments were carried out under physiological pH and temperature conditions. In order to initially determine an optimal metal concentration, hFF‐K4H and hFF‐K4H‐K25H (1.65 mM) were incubated at varying concentrations of copper ions over 20 h at 37°C, followed by rheology (Figures S9 and S10). For both peptides, we found that the most stable hydrogel resulted from a metal concentration of 0.25 equiv. per histidine residue. Therefore, all CD and rheology experiments described below were carried out under these conditions.

In order to clarify the role of histidine in metal coordination in this model, the three histidine‐free sequences hFF, hFF‐GA‐Man, and hFF‐GA‐Gal were first studied as negative controls. The addition of metal cations showed no impact on the structure of the parent aglycon sequence (Figure S1). A similar result was observed in the hFF‐GA‐Man (Figure S2). In the case of hFF‐GA‐Gal (Figure S3), the addition of divalent copper ions leads to an increase in the ratio between the intensity of the 208 nm minimum and that of the 220 nm minimum; that is, the 208 nm band appears to be “growing in”, indicating a decrease in the formation of higher ordered structures [32]. To summarize, metal ions appear to hardly influence overall peptide structure in the absence of histidine, except when galactose and copper are both present, indicating some interaction between them that leads to more typical alpha helical character and less higher ordered structure formation.

Moving on to the peptide of type hFF‐K4H, higher ordered structures (low 208 nm/220 nm ratio) are observed across the board in the absence of metal ions (Figure 2 and Figures S4 and S5). In the case of hFF‐K4H‐GA‐Gal, these higher order structures persist regardless of the nature of the divalent metal added (Figure S5). The most pronounced deviations from these higher order structures were observed when divalent copper ions were added to the glycan‐free (red trace in Figure S4) or to the mannose‐functionalized hFF‐K4H sequence (red trace in Figure 2A), with the latter change being most evident based on the strong intensity of the 208 nm band. To a lesser extent than copper, the divalent metals calcium, zinc, and iron also lead to a growing in of the 208 nm minimum in the mannose sample, likewise indicating reduced higher order structure formation in these cases (respective traces in Figure 2A).

For the subset of peptides with His at position 25, the CD data revealed significantly differential global conformation compared to the above‐described position 4 case (Figure 2B and Figures S6–S7). The non‐glycosylated parent shows somewhat higher order structure formation of the kind described above in the absence of metal and in the presence of metals calcium and iron, whereas loss of higher order structure is indicated by a growing in of the 208 nm band in the presence of copper, and a non‐assignable type of CD trace is observed when zinc (dark blue trace) is present (Figure S6). In the case of peptide hFF‐K25H‐GA‐Gal, more typical alpha‐helical signatures are observed in the absence of metal, and in the presence of metals zinc, iron, and copper, as indicated by the appreciable intensity of the 208 nm band, but again a non‐assignable conformation is indicated in the presence of calcium (pink trace in Figure S7). The hFF‐K25H‐GA‐Man peptide shows no evidence of higher ordered structure formation, but rather alpha‐helical character in the presence of copper and iron and an unassignable conformation in the absence of metal and in the presence of zinc or calcium. Overall, samples from this series show virtually no generalizable behavior. The α‐helical atypical, or unassignable, CD trace observed in the five cases given above is comparable to the one derived from a theoretical model from 1988 that determined the effects on CD of geometric distortions due to changes in the angle of the carbonyl group in the peptide backbone within regular secondary structures [33]. The change in position of the histidine from near the N‐terminus to near the C‐terminus can also lead to a possible interaction between the imidazole ring and the carboxy group of the C‐terminus [34] however, in order to determine the molecular basis of the unassignable traces, high‐resolution structural data would be needed, and these studies are ongoing.

Structural analysis of the doubly histidine‐substituted aglycon and glycopeptides of the hFF‐K4H‐K25H series was performed at pH 7 (Figures 3 and S8). The reduction in pH values from 7.4 of the prior data sets was necessary because the double‐His peptides precipitated at pH values 7.4 and above. The double‐His‐substituted aglycon peptide exhibits higher ordered structures except when divalent calcium ions are added, in which case the formation of more ideal α‐helical structures is indicated by the increase in ratio between the minimum at 208 and that at 222 nm (Figure S8). In the case of hFF‐K4H‐K25H‐GA‐Man (Figure 3A), the peptide shows the formation of more ideal alpha‐helical character in the absence of divalent metal ions, whereas the addition of Cu^2+^ and Zn^2+^ leads to the formation of higher ordered structures. In comparison, the hFF‐K4H‐K25H‐GA‐Gal (Figure 3B) shows the increase in ratio between the two minima (208 nm:222 nm), away from higher order structures, upon the addition of divalent metal cations.

In general, CD‐based analysis reveals that the His‐position is a crucial factor in the structure formation of hFF‐based peptides and the presence of metal ions tends to support alpha‐helical elements. In addition, the nature of the divalent ion has a significant effect on the peptide self‐assembly; interestingly, copper was found to have the most dramatic impact. Whereas both the variants of hFF‐K4H and hFF‐K4H‐K25H show comparable structural behavior, hFF‐K25H is of interest for further structural studies due to its atypical CD pattern.

### 3.3 Rheological Properties

The viscoelastic properties of the peptides were investigated using oscillatory shear rheology. For this purpose, peptide concentrations of 0.5 wt% were used, analogous to earlier work by Hellmund on hFF03 [19].

The peptide samples were subjected to an oscillating shear strain, and this experiment yields the storage modulus G′ (elastic response) and loss modulus G″ (viscous response) as a function of frequency. All individual frequency sweep results are shown in Figures S11–S22.

The viscoelastic properties of natural mucus generally vary depending on the organ in question and its state of health or disease. For example, healthy lung mucus has a storage modulus (G′) of approximately 1–10 Pa at a frequency higher than 1 rad/s [35]. The analysis of these properties of the hFF‐His series should provide information on whether thickening, liquefaction, or even no change is induced by the presence of metal ions.

In order to enable comparison of all measurements, the data recorded in the oscillatory rheology (Figures S11–S22) were summarized by plotting all storage moduli (G′ [Pa]) at a frequency of 67.7 rad/s, as shown in Figure 4. This choice of frequency was made due to our interest in the behavior of the hydrogels at higher frequency regions.

**FIGURE 4:**
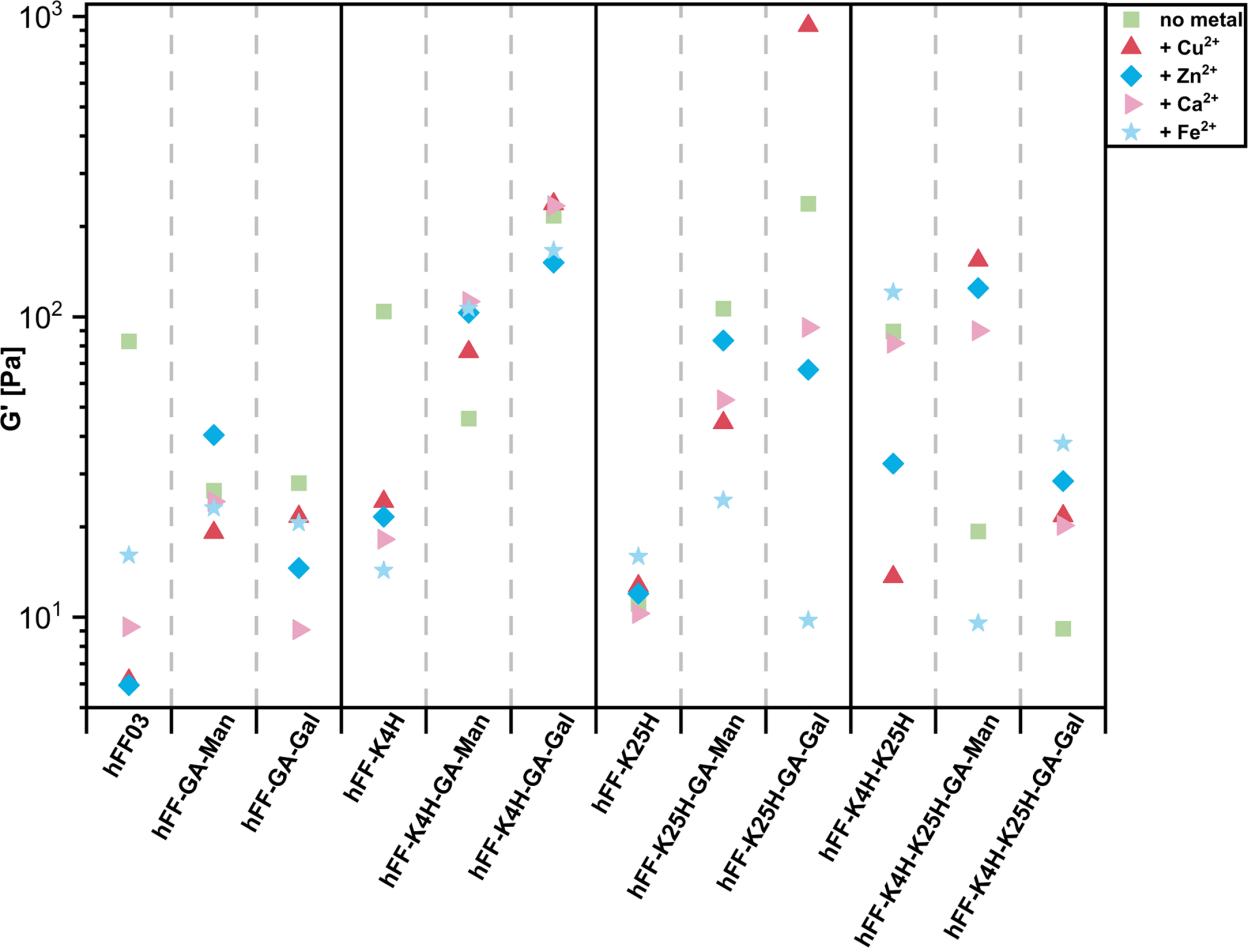
Summary of all storage moduli obtained by means of oscillatory rheology at a frequency of 67.7 rad/s. All raw data are available in Figures S11–S22.

Analysis of the viscoelastic properties of the metal‐free peptides revealed that neither the introduction of His at position 4 (hFF‐K4H) nor the introduction of two histidines into the scaffold (hFF‐K4H‐K25H) affects the stiffness of the hydrogel in comparison with hFF03. In contrast, the introduction of histidine at position 25 (hFF‐K25H) leads to a strong reduction in the storage modulus, by about 80%. The proximity to the C‐terminus of position 25 could play a decisive role here, because the C‐ and N‐functionalization analyzed by Heinz et al. [31] may be prevented by possible π‐stacking of His and [2]‐Abz‐OH [36].

Modifications with glycans at position 18 show an influence on the viscoelastic properties. Peptide hFF03 shows similar reductions in the viscoelasticity when mannose or galactose is introduced. The influence of glycan is differential when analyzing the hFF‐K4H and hFF‐K25H series. The mannose‐substituted peptide (hFF‐K4H‐GA‐Man) shows the formation of a softer hydrogel, whereas the galactose‐substituted peptide (hFF‐K4H‐GA‐Gal) forms a stiffer hydrogel than hFF‐K4H. Glycan‐substituted hFF‐K25H samples show strong stiffening of the hydrogel, whereby hFF‐K25H‐GA‐Gal with its storage modulus of 238 Pa is twice as stiff as hFF‐K25H‐GA‐Man. Compared to hFF‐K25H, the His‐disubstituted peptide (hFF‐K4H‐K25H) forms softer hydrogels with the incorporation of both glycans.

The addition of metal cations in a ratio of 0.25 equiv. per His residue has an impact on the viscoelastic properties of the hydrogels. No general trend can be observed across variants, but the results are dramatic in certain individual samples. Potentially, the absence of possible coordination sites in the hFF03 peptide leads to the decrease in viscoelasticity with the addition of divalent metal ions in all variants, except for hFF‐GA‐Man + Zn^2+^ (Figures S11–S13).

Unexpectedly, in the case of hFF‐K4H with its single coordination site, the addition of all four divalent metal ions shows approximately the same reduction in G′, whereas the stiffness of hFF‐K4H‐GA‐Man increases with the addition of all four metal ions (Figure 4 and Figures S14–S16). It was observed that Cu^2+^ has the least rigid hydrogel of the divalent metal ions (Figure 4). Meanwhile, the galactose‐substituted hFF‐K4H shows the stiffest hydrogel of the hFF‐K4H and its derivatives in the presence of Cu^2+^. Similarly, hFF‐K4H‐GA‐Gal + Ca^2+^ shows appreciable stiffness.

In the case of position 25, the glycan‐free hFF‐K25H shows no significant changes in the storage modulus at a frequency of 67.7 rad/s due to the addition of divalent metal ions (Figure 4 and Figures S17–S19). Similar to the behavior described above for position 4, hFF‐K25H‐GA‐Gal + Cu^2+^ is found to be a stiffer hydrogel that those obtained by adding another divalent metal ion. This even more pronounced reduction in comparison to hFF‐K4H (∆G′ (hFF‐K25H‐GA‐Gal presence and absence of Fe^2+^) = 228 Pa) and its derivatives can be explained by the proximity of the histidine to the glycan. While hFF‐K4H is two heptads away from the glycan, hFF‐K25H is only one. As metal coordination can occur not only at the histidine, but also at the hydroxyl groups of glycans, coordination with the divalent metal ion can occur due to the proximity to the glycan. This in turn leads to the interruption of possible hydrogen bonds of the glycan and the associated reduction of the viscoelastic properties. The orientation of the hydroxyl group also plays a role in the possibility of coordination [37]. The different storage moduli, due to the combination of different metal ions and glycan, can be attributed to the different properties of the divalent metal ions and their coordination possibilities. However, the mannose and galactose derivatives of hFF‐K25H show no trend that could explain possible properties.

The introduction of a second histidine into the peptide (hFF‐K4H‐K25H) in the case of glycan‐substituted peptides by adding divalent metal ions shows the stiffening of the hydrogels by increasing storage modulus, except for hFF‐K4H‐K25H‐GA‐Gal + Fe^2+^ (Figure 4 and Figures S20–S22). This stiffening of the hydrogels may be due to the incorporation of an additional coordination site. In the case of hFF‐K4H‐K25H, the divalent metal ions tend to have a negative influence on viscoelastic properties. One possible explanation for the enhanced stiffness of the glycosylated analogues is that the introduction of a second coordination possibility whereby the divalent metal ions preferentially coordinate with the histidine and are unavailable for possible coordination with the hydroxy groups of the glycans.

Overall, the data collected by means of oscillatory rheology do not point to any clear trend; however, certain summary statements can be made. First, in the absence of monosaccharide and metal, single substitution of lysine with histidine at position 25 leads to compromised viscoelastic properties, whereas substitution at either position 4 alone or disubstitution does not compromise viscoelastic properties. Second, across the board, peptides attached with monosaccharide display less favorable viscoelastic properties in the presence of metal ions than in the absence. Third, the delicate interplay between histidine position and identity of the monosaccharide and divalent metal is demonstrated best by two examples: (a) hFF‐K25H‐GA‐Gal + Cu^2+^, which represents the most dramatic case in which metal ions boosted viscoelastic properties within the series, and (b) in general, histidine at position 4 attached with mannose leads to worsened viscoelastic properties compared to galactose.

### 3.4 Transmission Electron Microscopy (cryo‐TEM)

**FIGURE 5:**
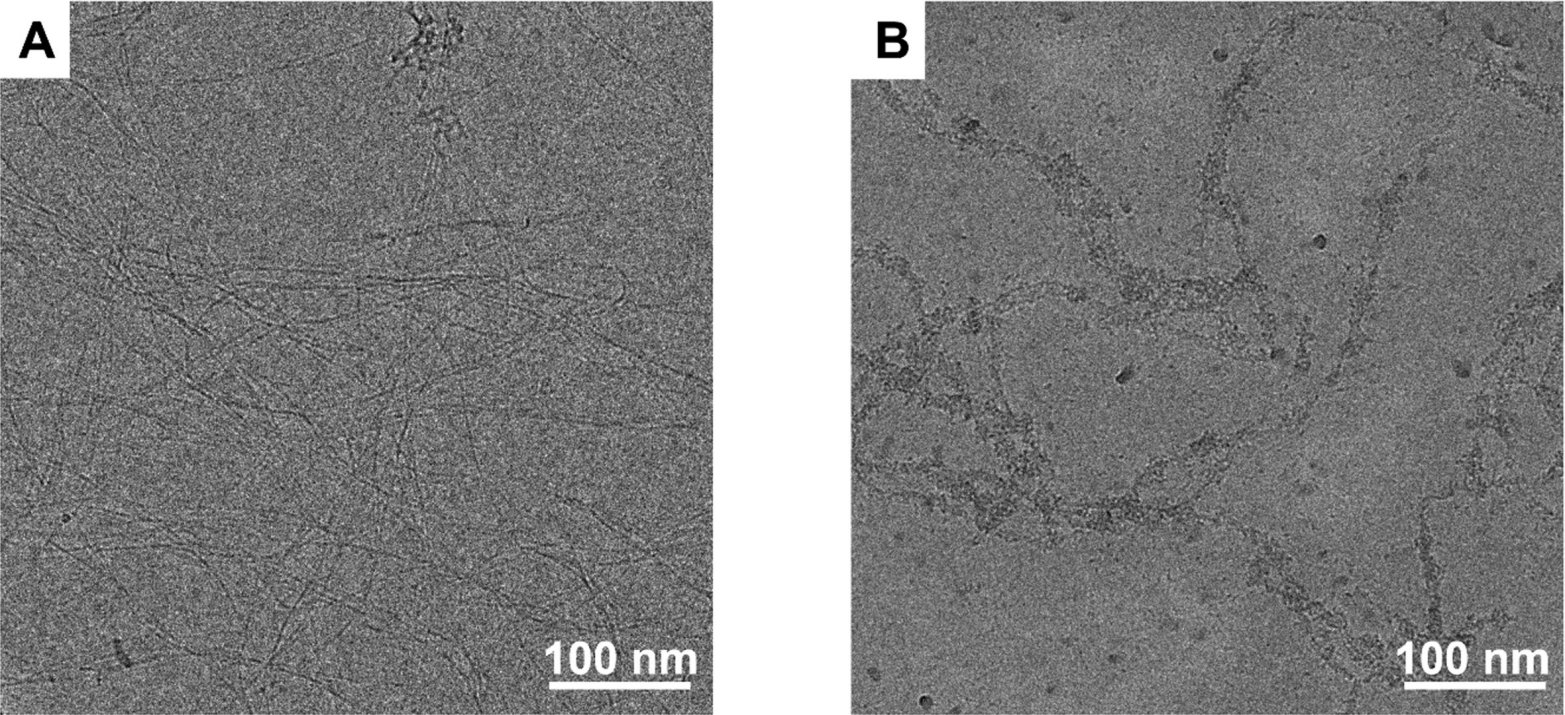
Crops from representative cryo‐TEM images of 0.1 mM peptide hydrogels. (A) hFF‐K25H‐GA‐Gal. (B) hFF‐K25H‐GA‐Gal and 0.25 equiv. Cu^2+^.

Due to the highly favorable viscoelastic properties of hFF‐K25H‐GA‐Gal (Figure S19) and its significant change in CD signal upon the addition of copper ions (red trace in Figure S7), this system appeared to be particularly suitable for further microscopic structure investigation with cryo‐TEM. Samples were prepared at concentrations of 0.1 mM in water at pH 7.4, in metal ion‐free water (Figures 5A and S27) and in the presence of 0.25 equiv. divalent copper ions (Figures 5B and S28). Figure 5A shows the formation of long fibers for hFF‐K25H‐GA‐Gal, as had already been found for the mannose‐glycosylated peptides of hFF03 [19, 20], demonstrating that substitution of lysine by histidine at position 25 into the hFF03 scaffold does not cause any observable morphological changes. The addition of 0.25 equivalents of divalent copper ions to hFF‐K25H‐GA‐Gal leads to a dramatically different morphology: densely packed fiber bundles with undefined thickenings are observed (Figure 5B). Such agglomerated structures were not present in the copper‐free samples. Although the TEM micrographs do not provide conclusive information on the source of the particular structural changes (H‐bonding or copper complexation), they clearly prove a morphological difference upon copper addition to hFF‐K25H‐GA‐Gal, in consistency with the CD and rheological data. Dark spots that appear in and along the fiber bundles support our idea of metal ion clusters bonding the individual fibers together. It could be imagined that the observed agglomeration could mimic natural mucus, especially if in bulk and at high concentrations. The microscopic structures of hFF‐K4H‐GA‐Man and hFF‐K4H‐Gal in the absence and presence of copper ions were likewise examined by cryo‐TEM (Figures S23–S26). Metal‐free (Figures S23 and S25) and copper‐containing (Figures S24 and S26) hFF‐K4H‐GA‐Man and hFF‐K4H‐Gal samples display fibril structures similar to those observed for metal‐free hFF‐K25H‐GA‐Gal discussed above; therefore, neither substitution of lysine for histidine at position 4 nor the presence of copper ions leads to any observable morphological change. This result further underlines the dramatic responsiveness, that is, agglomeration, of the hFF‐K25H‐GA‐Gal peptide in the presence of divalent copper ions.

## 4 Conclusion

In this work, glycopeptide hydrogels were designed as chemically precisely defined models for the purpose of determining the effects of glycosylation and histidine‐mediated divalent metal coordination on the structure and viscoelastic properties. Glycopeptide conformation was analyzed under different yet naturally relevant conditions by means of CD spectroscopy and TEM, and viscoelastic properties were investigated by rheology. As a result, the combination of metal complexing functionalities, the nature of the metal ion (with different coordination spheres), and the nature and position of glycosylation create a complex situation with dramatic effects on the viscoelastic properties of nature‐inspired hydrogels. To provide a navigable overview, all results obtained in this study are provided in Table 2.

**TABLE 2:** Overview of all results obtained in this study.

Abbreviation	Structure, viscoelastic properties, morphology
*hFF	Higher ordered structures, no change with divalent cations Decrease in viscoelastic properties with addition of divalent cations
hFF‐GA‐Man	Higher ordered structures, no change with divalent cations Decrease in viscoelastic properties with addition of divalent cations
hFF‐GA‐Gal	Higher ordered structures, more ideal α‐helical character with Cu^2+^ Decrease in viscoelastic properties with addition of divalent cations
hFF‐K4H	Higher ordered structures, more ideal α‐helical character with Cu^2+^ Decrease in viscoelastic properties with addition of divalent cations
hFF‐K4H‐GA‐Man	Higher ordered structures, more ideal α‐helical character with divalent cations (Fe^2+^ < Zn^2+^ < Ca^2+^ < Cu^2+^) Increase viscoelastic properties with addition of divalent cations Fibril formation, no morphological change with addition on 0.25 equiv. Cu^2+^
hFF‐K4H‐GA‐Gal	Higher ordered structures, no change with divalent cations Decrease in viscoelastic properties with Zn^2+^ and Fe^2+^ Increase in viscoelastic properties with Cu^2+^ and Ca^2+^ Fibril formation, no morphological change with addition on 0.25 equiv. Cu^2+^
hFF‐K25H	Higher ordered structure, more ideal α‐helical character with Cu^2+^, unassignable structure with Zn^2+^ Decrease in viscoelastic properties with Cu^2+^ and Zn^2+^ No change in viscoelastic properties with Fe^2+^ and Ca^2+^
hFF‐K25H‐GA‐Man	More ideal α‐helical character with Cu^2+^ and Fe^2+^, unassignable structure without divalent metal ion and with Zn^2+^ and Ca^2+^ Decrease in viscoelastic properties with divalent metal ions (Fe^2+^ > Cu^2+^ > Ca^2+^ > Zn^2+^)
hFF‐K25H‐GA‐Gal	More ideal α‐helical character with Zn^2+^, Fe^2+^ and Cu^2+^, unassignable structure with Ca^2+^ Highest viscoelastic properties with Cu^2+^ and lowest viscoelastic properties with Fe^2+^ of whole hFF‐His series
hFF‐K4H‐K25H	Higher ordered structures, more ideal α‐helical character Ca^2+^ Decrease in viscoelastic properties with Cu^2+^ and Zn^2+^ No change in viscoelastic properties with Fe^2+^ and Ca^2+^
hFF‐K4H‐K25H‐GA‐Man	Higher ordered structures, no change with divalent cations Increase in viscoelastic properties with Cu^2+^, Zn^2+^ and Ca^2+^, no change with Fe^2+^
hFF‐K4H‐K25H‐GA‐Gal	More ideal α‐helical character (Cu^2+^ < Zn^2+^ < Fe^2+^ < Ca^2+^) Increase in viscoelastic properties with Ca^2+^, Zn^2+^ and Fe^2+^, no change with Cu^2+^

CD data revealed that metal‐free glycopeptides, already at a concentration of 0.5 wt%, formed higher ordered structures, which were comparable to those of the parent peptide hFF03, whereas the addition of the divalent metal cations to the His‐containing variants of hFF03 showed changes in structure and rheology. Peptides hFF‐K4H and hFF‐K4H‐K25H and their glycosylated variants showed varying structures, including α‐helix or higher ordered structures, in the presence and absence of divalent metal ions. In the case of hFF‐K25H, α‐helical atypical CD spectra were observed, which were comparable with a theoretical model from 1988 [33]. Addition of Cu^2+^ and Fe^2+^ shows stabilization of the typically α‐helical structure, whereas for Zn^2+^ and Ca^2+^, the unusual conformation persists.

Rheology experiments showed that all peptides of the hFF‐His library investigated at physiological pH formed hydrogels. The positions of the histidine within the hFF‐based peptide were shown to play a key role in influencing the viscoelastic properties on interaction with divalent metal cations. The incorporation of His at position 4 (hFF‐K4H) led to stronger hydrogels compared to the incorporation of His in position 25 (hFF‐K25H). The insertion of two histidine residues per peptide leading to hFF‐K4H‐K25H showed a stronger increase in their viscoelastic properties that was, however, weakened through the introduction of either glycans. It was found that the addition of divalent metal ions showed different effects on the viscoelastic properties of the hydrogels. Although the glycopeptides of hFF‐K4H were little affected to the same extent, the glycopeptides of hFF‐K25H showed that the divalent metal ions individually influenced these properties. This may be due to the position and proximity to the C‐ or N‐terminus of the histidine in the peptide. Only for the case of introducing a second coordination partner into the peptide (hFF‐K4H‐K25H) one observe more generally a stiffening of the hydrogel by the addition of divalent metal ions in its glyco‐derivatives. Somewhat surprisingly, double substitution of lysine with histidine did not lead to dramatic effects in overall viscoelastic properties; rather, these are comparable to the single substitution species in spite of the a priori design idea that each histidine represents an excellent ligand for metal binding.

Analysis by cryo‐TEM of hFF‐K25H‐GA‐Gal showed that the peptide forms thick fibril bundles with divalent copper ions. This result is comparable to the rheological data, where hFF‐K25H‐GA‐Gal in the presence of divalent copper ions shows the highest viscoelasticity of the hFF‐His series.

The analysis of the His‐containing glycopeptides and their comparison to the His‐free variants carried out in this work impressively shows the key players driving hydrogel formation: the nature of the divalent metal ion, glycan position, and His position in the scaffold. This can be observed by the reduction of the stiffness upon the change in position of Histidine from 4th to 25th position. However, the combination of galactose, histidine at position 25, and the addition of Cu^2+^ showed the formation of the most viscous hydrogel in the complete series of measurements. Despite the fact that hFF‐K25H is the weakest hydrogel, this sequence is of high interest due to the broad range of observations made in the presence and absence of both glycans and divalent metals; that is, it can be considered a highly responsive sequence. Also, of interest for further study is the disubstituted hFF‐K4H‐K25H, which is unique in its generally increased gel behavior upon metal addition.

The results presented in this manuscript show that the divalent metal cations significantly influence the properties of glycopeptide‐based hydrogels. The data presented here serve as a foundation for the development and study of further glycopeptide‐histidine‐containing peptides with the aim to gain further insight into design principles of stimulus‐responsive biomimetic hydrogels.

## Conflicts of Interest

The authors declare no conflicts of interest.

## Supporting information


**Data S1:** Supporting Information.

## Data Availability

All data are made available in the Supporting Information.
