# Supplementary material for: Metal Ion–Induced Cross‐Linking in Mucin‐Inspired Peptide Hydrogels
Source: J Pept Sci. 2025 Sep 23;31(11):e70059. doi: 10.1002/psc.70059 (PMC12457099; doi:10.1002/psc.70059)
Supplement: Supplementary file 1 — Data S1: Supporting Information. [file PSC-31-e70059-s001.pdf]

Supporting information

Metal-ion induced cross-linking in mucin-inspired peptide hydrogels

Annelie Puhlmann[1a)], Cihan Baydaroglu[2], Boris Schade[1b)], Michael Gradzielski[2] and Beate Koksch[1a),*]

1a) Institute of Chemistry and Biochemistry, Freie Universität Berlin, Arnimallee 20, 14195 Berlin, Germany

b) Research Center of Electron Microscopy, Freie Universität Berlin, Fabeckstraße 36A, 14195 Berlin, Germany

2 Stranski-Laboratorium für Physikalische und Theoretische Chemie, Institut für Chemie, Technische Universität Berlin, Straße des 17. Juni 124, 10623 Berlin, Germany

* Author to whom correspondence should be addressed.

Contents

1. Procedure 2
   1.1. Synthesis of mucin-inspired peptides 2
   1.2. Sample preparation 3
2. Rheology (S9 – S22) 19
3. Transmission electron microscopy (TEM) 22
5. References 27

1. Procedure

1.1. Synthesis of mucin-inspired peptides

Peptide synthesis

All peptide syntheses were carried out using Liberty Blue TM (CEM corporation, Matthews, NC, USA) microwave-assisted peptide synthesizer. Using Oxyma/1,3-diisopropylcarbodiimide (DIC) as activating agents the synthesis was performed at 0.1 mmol scale. Full length peptides were synthesized on Fmoc-Leu-NovaSyn TGA (0.24 mmol/g loading) resin from Novabiochem. A 5-fold excess of amino acid (Carbolution) was used in each coupling. Glutamic acid was protected by *tert*-butyl ester (*t*Bu), histidine by *tert*-butyl ester (*t*Bu) and lysine by *tert*-butoxycarbonyl (Boc), except for the lysine incorporated at position 18 which was protected by monomethoxytrityl (Mmt) (purchased from CEM) for orthogonal deprotection and subsequent functionalization. Undecorated hFF peptide was cleaved from the resin by treatment with a mixture of trifluoroacetic acid (TFA) and triisopropylsilane (TIPS) (95: 5 v/v; 5 mL/g of resin) for 3 h at room temperature.

Glycan decoration

Glycan decoration was performed at the 18th position in the peptide chain were Lysin was orthogonal protected with a Mmt-protecting group. Deprotection of Mmt was conducted using a cleavage cocktail with DCM/TFE/AcOH (7:2:1). First 5 mL were added and agitated for 1 hour. Afterwards a new solution of this cleavage cocktail was used to deprotect for another 6 hours. The solution was removed and the resin washed three times with DCM. The resin was suspended in DMF and a solution of glutaric anhydride (10 equiv.) with DIPEA (20 µL) in DMF were added. The mixture was agitated overnight and afterwards washed three times each with DMF and DCM. Mmt cleavage and glutaric anhydride coupling were repeated once. In an Eppendorf vial (2 mL) was 3 equiv. of the amidated glycan added and solved in DMF in the ultrasound bath. Another Eppendorf vial was used to dissolve COMU (3 equiv.) in DMF. The resin was suspended in DMF for 30 min and the COMU solution with DIPEA (6 equiv.) were added. The reaction was agitated in the ultrasound bath at 40°C for 1 min, the amidated glycan added and agitated for 1.5 hours. Afterwards the resin was washed with DMF and DCM.

Cleavage of Resin was performed using cleavage cocktail of 45% DCM, 50% TFA (v/v) and 5% TIPS (v/v) for 1.5 hours. The cleaved peptide was transferred to round bottom flask and the TFA and DCM were evaporated. Peptide was precipitated in cold ether and the solution centrifugated in a Falcon tube (15 mL). The ether was decanted, and the crude peptide redissolved in MilliQ-water and lyophilized overnight.

Lypohilization of synthesized compounds was performed with a laboratory freeze dryer ALPHA 1-2 LC (Christ Gefriertrocknungsanlagen GmbH, Osterode im Harz, Germany) which was connected to a chemistry hybrid pump RC 6 (Vacuubrand GmbH + Co KG, Wertheim, Germany).

Purification and characterization

Analytical HPLC was performed on a VWR-Hitachi Chromaster HPLC 600 bar system (VWR International GmbH, Darmstadt, Germany). Separation was performed using a preparative C_{18}-column (Kinetex® 5 µm C18 100 Å, LC Column size: 250 x 4.6 mm) and the eluents water (solvent A) and acetonitrile (solvent B), both containing 0.1% (v/v) TFA.

Purification was performed using a LaPrep∑HPLC system (VWR International GmbH, Darmstadt, Germany). Prior filtration of dissolved peptides was performed through a 25 mm filter with a 0.45 µm PTFE membrane (Chromafil® Xtra). Separation was performed using a preparative C_{18}-column (Kinetex® 5 µm C18 100 Å, LC Column size: 250 x 21.2 mm) and the eluents water (solvent A) and acetonitrile (solvent B), both containing 0.1% (v/v) TFA. Fractions were collected in test tubes and analyzed with analytical HPLC overnight. Pure fractions were merged, and acetonitrile removed using a rotatory evaporator. The remaining peptide in water was frozen with liquid nitrogen and dried by lyophilization.

Reaction controls and pure peptide were analyzed using mass spectrometry using LC-ESI-TOF: Agilent 6230 System with 1260 Infinity II LC system and 1100 DAD detector.

The TFA salts of the purified peptides were exchanged with HCl by dissolving the peptides in 4mL MilliQ-water followed by the addition of 6M HCl (3.75 µL/5 mg peptide) and subsequent lyophilization. This procedure was repeated four times.

1.2. Sample preparation

Peptide stock solution

Peptide stock solutions were prepared in a 15 mL Falcon tube by dissolving the dried peptide powder in HFIP or water (1 mL).

Determinations of the stock solutions concentrations of peptides containing Abz as N-terminal labels were completed by measuring the absorbance of H-*o*Abz-Gly-OH (λ_{max} = 320 nm, pH 7.4) in buffer (PBS + 6 M GndHCl). A calibration curve was used to calculate the concentration of the stock solution.[1]
Preparation of UV-Vis-samples was accomplished converting 10 µL from the stock solution into three different 1.5 mL Eppendorf vials. To minimize the pipetting error three sample of the same stock solution were prepared. HFIP was evaporated using a gentle stream of nitrogen and the residue was redissolved in 1 mL of D-PBS containing 6M Guanidine-Hydrochloride (pH 7.4). The UV-Vis measurements were performed with a semi-micro cell 104-QS, 10 mm cuvette of Hellma Analytics using 500 µL of the prepared solution. The absorbance (at λ = 320 nm) average of these three samples were used to determine the concentration of the stock solution.

Metal complexation experiments

From the stock solutions were 1.65 mM (~ 0.5 wt%) added into a vial. HFIP was evaporated using a gentle stream of nitrogen and the peptide was redissolved in water (100 µL (Rheology) and 30 µL (CD)). For the gel formation experiments with metal coordination a metal stock solution (0.1 M) in water (pH

7.4) was prepared and 0.25 equiv. Metal per Histidine was added to the solution. The volume was adjusted to a total volume of 150 µL (rheology) or 40 µL (CD) and vortexed for 10 seconds. All experiments were performed with an incubation time of 20 h at 37°C using Eppendorf ThermoMixer® C (Eppendorf, Hummelsbüttel).

Table S1: hFF

Abz LKKELAALKKELAALKKELAALKKEL

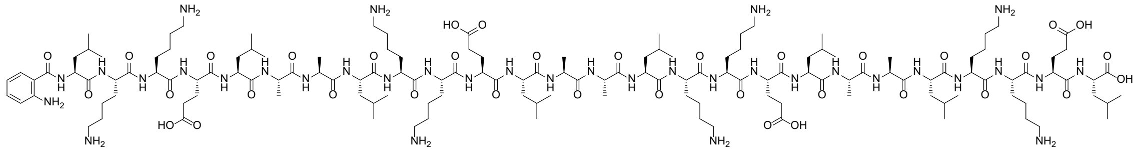

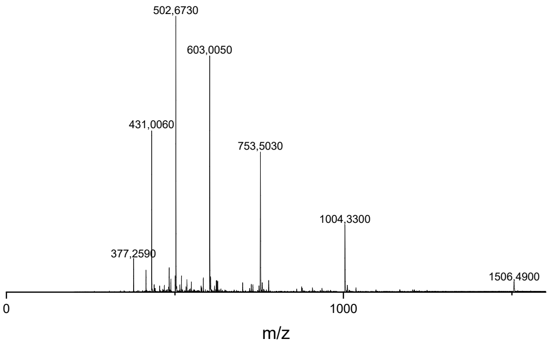

ESI-MS

	expected	received
$[M+2H]^{2+}$	1505.4365	1506.4900
$[M+3H]^{3+}$	1003.9576	1004.3300
$[M+4H]^{4+}$	753.2182	753.5030
$[M+5H]^{5+}$	602.7746	603.0050
$[M+6H]^{6+}$	502.4788	502.6730
$[M+7H]^{7+}$	430.8389	431.0060
$[M+8H]^{8+}$	377.1091	377.2590

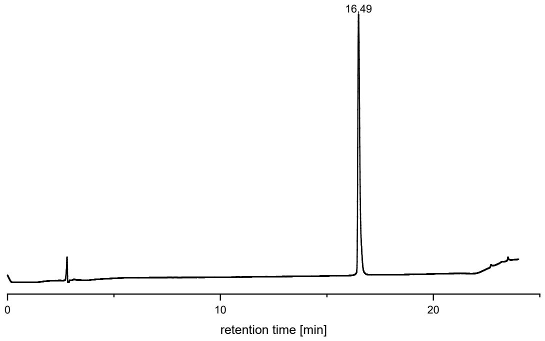

HPLC

10% ACN (0.1% TFA) to 60% ACN in Water (18 min)

retention time: 16.49 min

CD-concentration

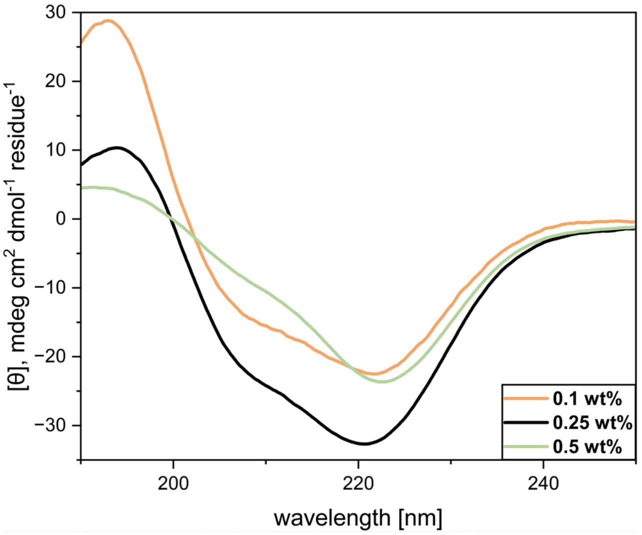

Table S2: hFF-GA-Man

Abz	LKKELAALKKELAALK**K**$_{(GA\text{-}Man)}$ELAALKKEL

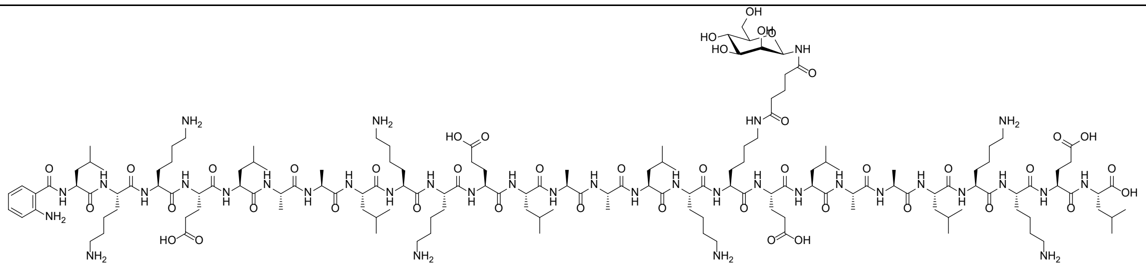

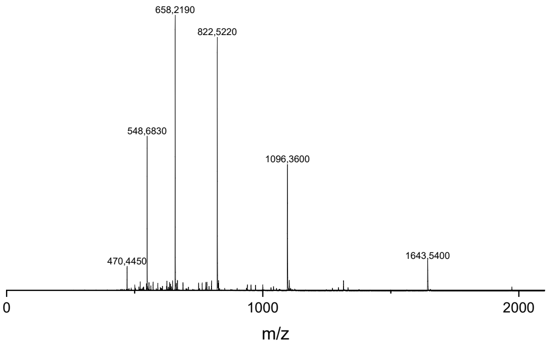

ESI-MS

	expected	received
$[M+2H]^{2+}$	1642.9868	1643.5400
$[M+3H]^{3+}$	1095.6578	1096.3600
$[M+4H]^{4+}$	821.9934	822.5220
$[M+5H]^{5+}$	657.7947	658.2190
$[M+6H]^{6+}$	548.3289	548.6830
$[M+7H]^{7+}$	470.1390	470.4450

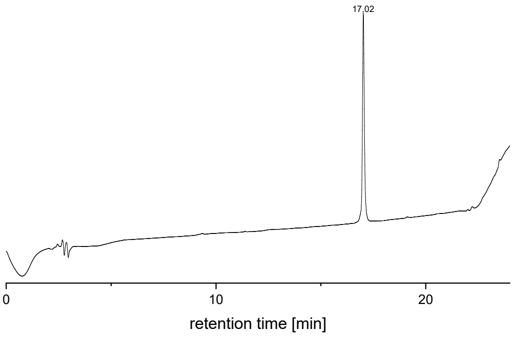

HPLC

10% ACN (0.1% TFA) to 60% ACN in Water (18 min)

retention time: 17.02 min

CD-concentration

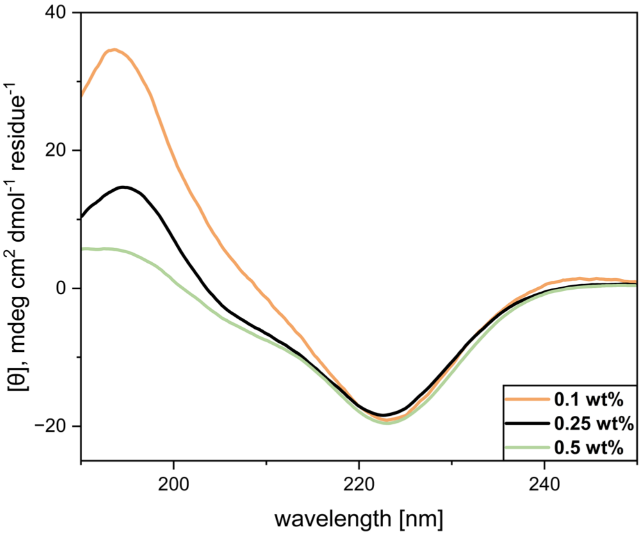

Table S3: hFF-GA-Gal

Abz LKKELAALKKELAALK**K**$_{(GA\text{-}Gal)}$ELAALKKEL

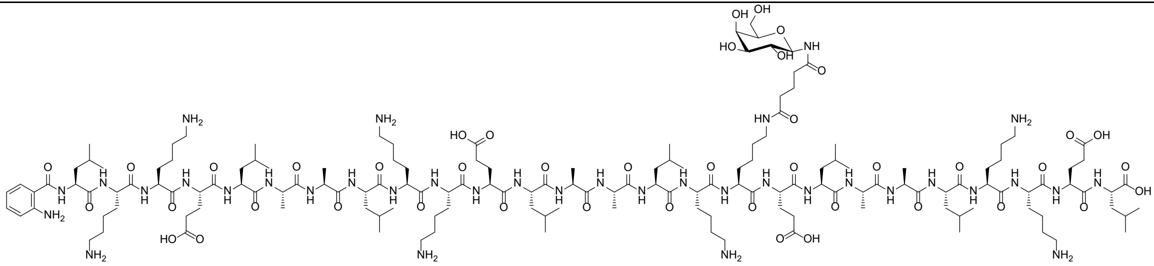

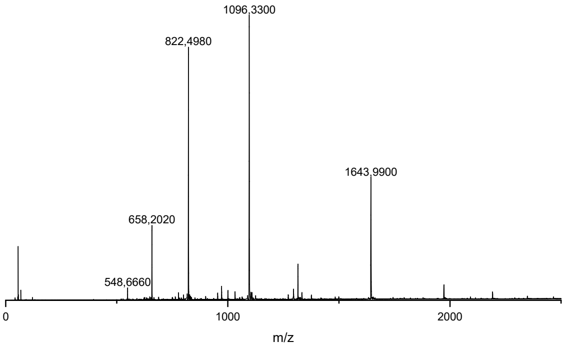

ESI-MS

	expected	received
$[M+2H]^{2+}$	1642.9868	1643.9900
$[M+3H]^{3+}$	1095.6578	1096.3300
$[M+4H]^{4+}$	821.9934	822.4980
$[M+5H]^{5+}$	657.7947	658.2020
$[M+6H]^{6+}$	548.3289	548.6660

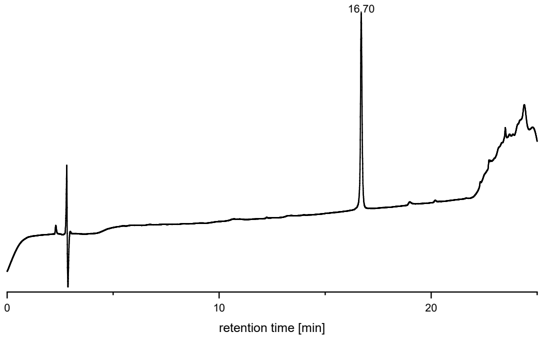

HPLC

10% ACN (0.1% TFA) to 60% ACN in Water (18 min)

retention time: 16.70 min

CD-concentration

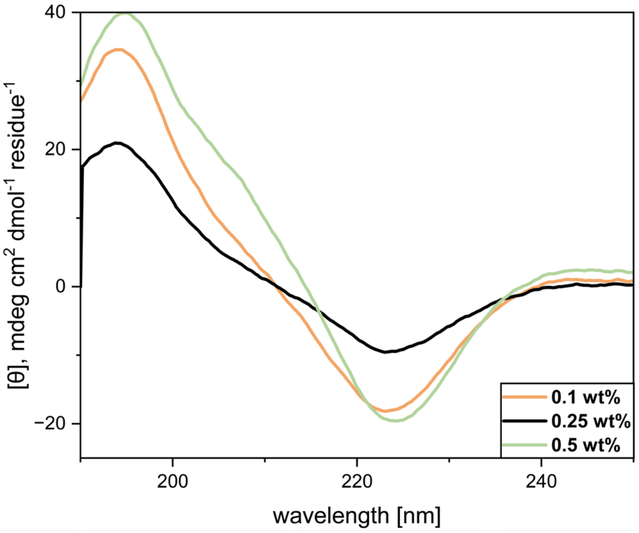

Table S4: hFF-K4H

Abz	LK**H**ELAALKKELAALKKELAALKKEL

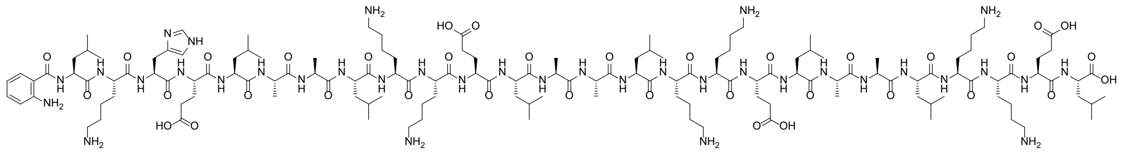

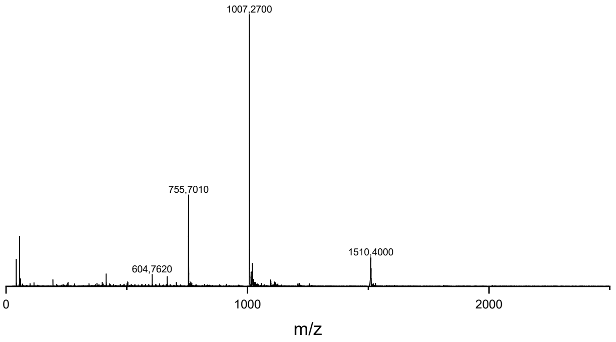

ESI-MS

	expected	received
$[M+2H]^{2+}$	1509.9185	1510.4000
$[M+3H]^{3+}$	1006.9456	1007.2700
$[M+4H]^{4+}$	755.4592	755.7010
$[M+5H]^{5+}$	604.5674	604.7620

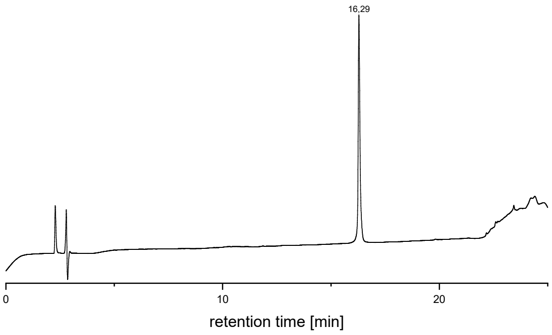

HPLC

10% ACN (0.1% TFA) to 60% ACN in Water (18 min)

retention time: 16.29 min

CD-concentration

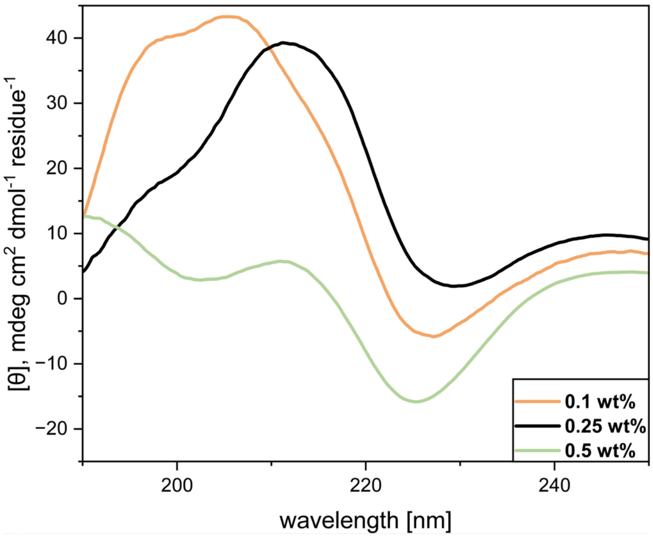

Table S5: hFF-K4H-GA-Man

Abz LK**H**ELAALKKELAALK**K**$_{\text{(GA-Man)}}$ELAALKKEL

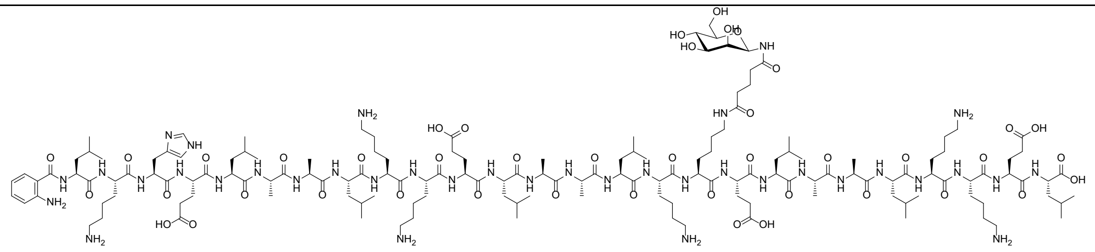

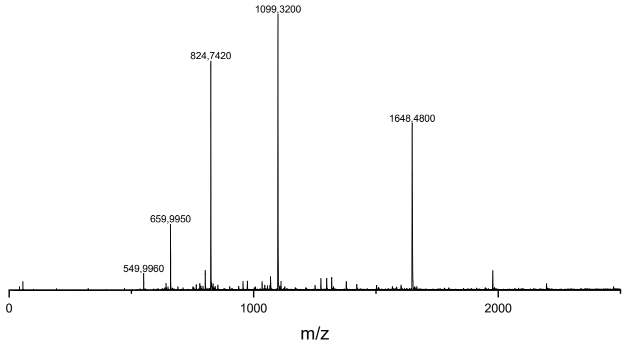

ESI-MS

	expected	received
$[M+2H]^{2+}$	1647.4687	1648.4800
$[M+3H]^{3+}$	1098.6458	1099.3200
$[M+4H]^{4+}$	824.2344	824.7410
$[M+5H]^{5+}$	659.3737	659.9960
$[M+6H]^{6+}$	549.8229	549.9980

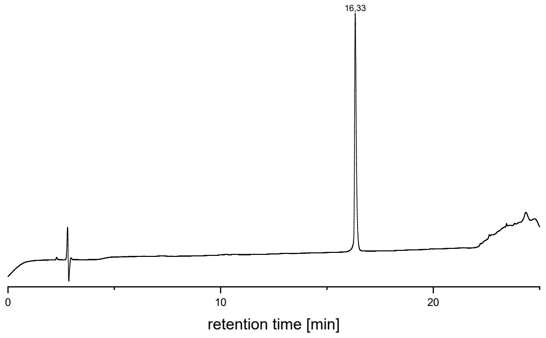

HPLC

10% ACN (0.1% TFA) to 60% ACN in Water (18 min)

retention time: 16.33 min

CD-concentration

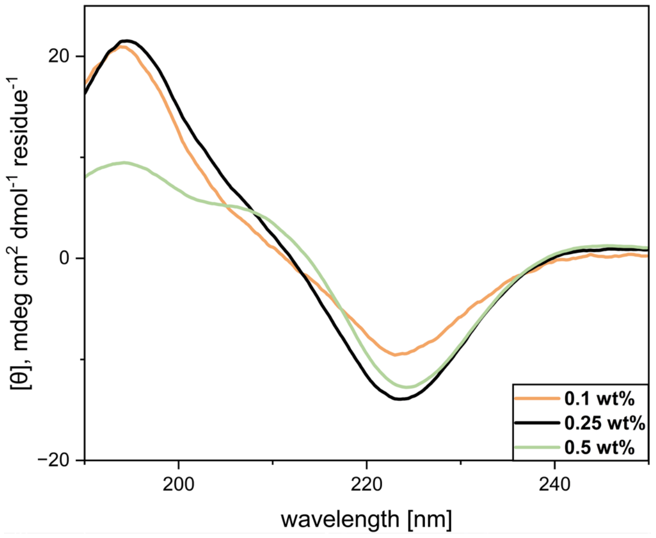

Table S6: hFF-K4H-GA-Gal

Abz LK**<u>H</u>**ELAALKKELAALK**K**$_{\text{(GA-Gal)}}$ELAALKKEL

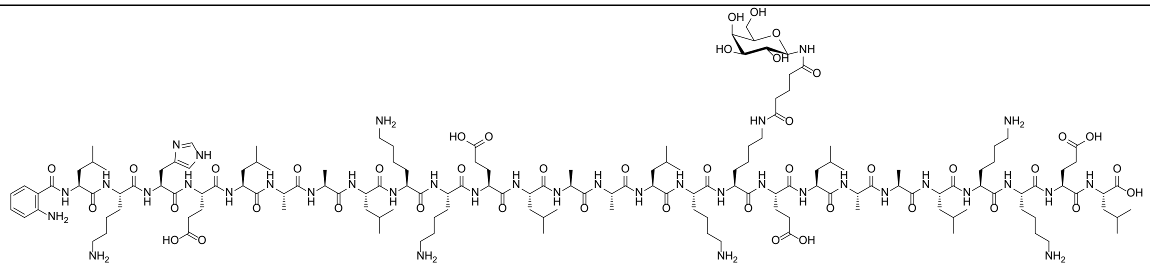

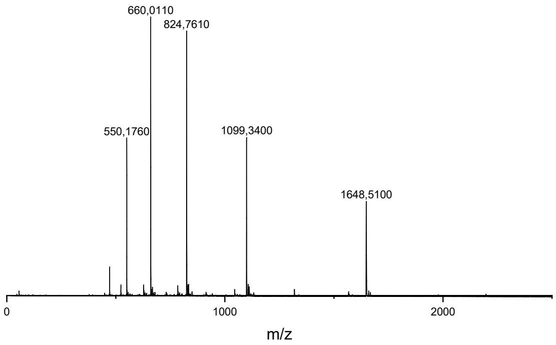

ESI-MS

	expected	received
$[M+2H]^{2+}$	1647.4687	1648.5100
$[M+3H]^{3+}$	1098.6458	1099.3400
$[M+4H]^{4+}$	824.2344	824.7610
$[M+5H]^{5+}$	659.5875	660.0110
$[M+6H]^{6+}$	549.8229	550.1760

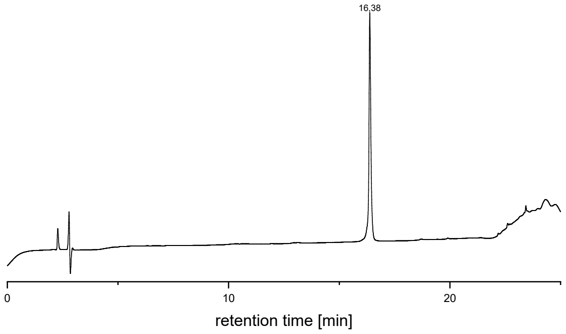

HPLC

10% ACN (0.1% TFA) to 60% ACN in Water (18 min)

retention time: 16.38 min

CD-concentration

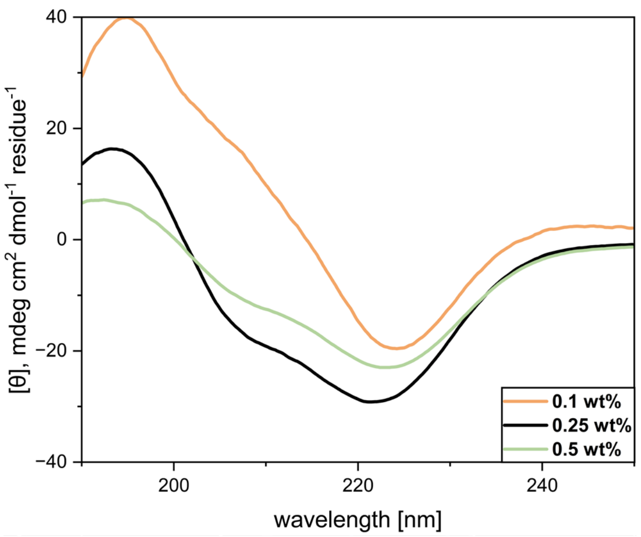

Table S7: hFF-K25H

Abz LKKELAALKKELAALKKELAALKHEL

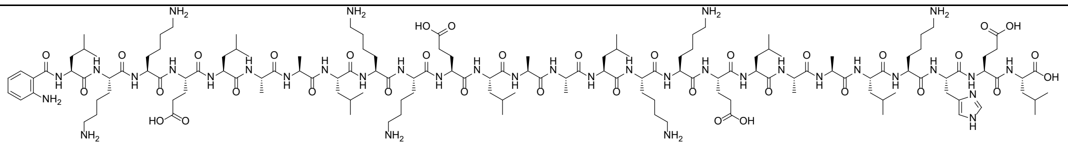

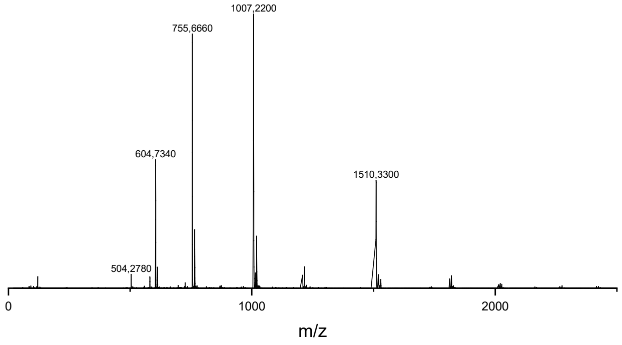

ESI-MS

	expected	received
$[M+2H]^{2+}$	1509.9185	1510.3300
$[M+3H]^{3+}$	1006.9456	1007.2200
$[M+4H]^{4+}$	755.4592	755.6660
$[M+5H]^{5+}$	604.5674	604.7340
$[M+6H]^{6+}$	503.9728	504.2780

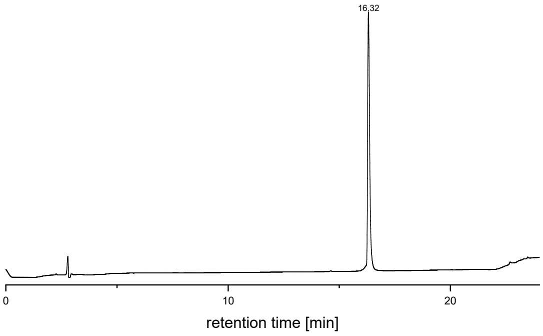

HPLC

10% ACN (0.1% TFA) to 60% ACN in Water (18 min)

retention time: 16.32 min

CD-concentration

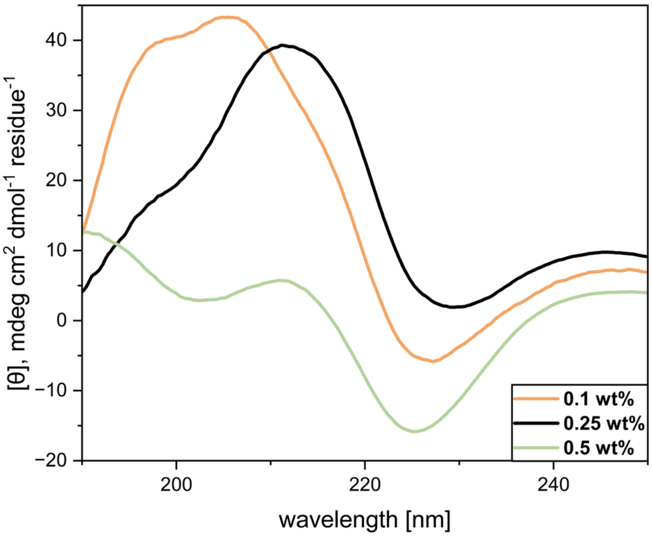

Table S8: hFF-K25H-GA-Man

Abz LKKELAALKKELAALK**K**(GA-Man)ELAALK<u>**H**</u>EL

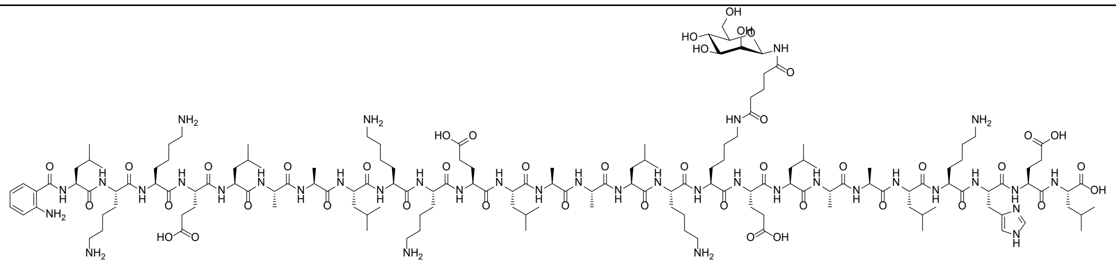

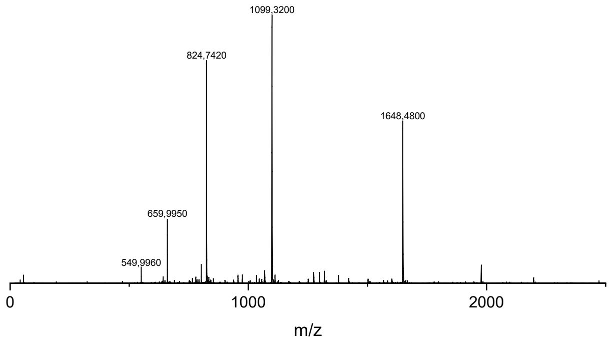

ESI-MS

	expected	received
$[M+2H]^{2+}$	1647.4687	1648.4800
$[M+3H]^{3+}$	1098.6458	1099.3200
$[M+4H]^{4+}$	824.2344	824.7420
$[M+5H]^{5+}$	659.5875	659.9950
$[M+6H]^{6+}$	549.8229	549.9960

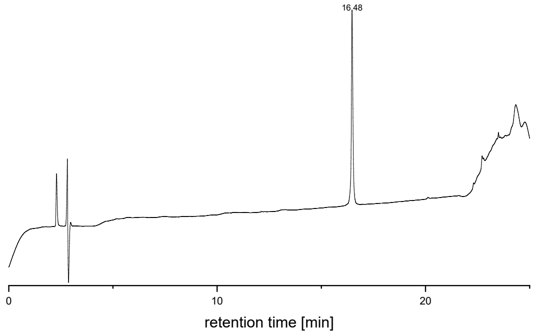

HPLC

10% ACN (0.1% TFA) to 60% ACN in Water (18 min)

retention time: 16.48 min

CD-concentration

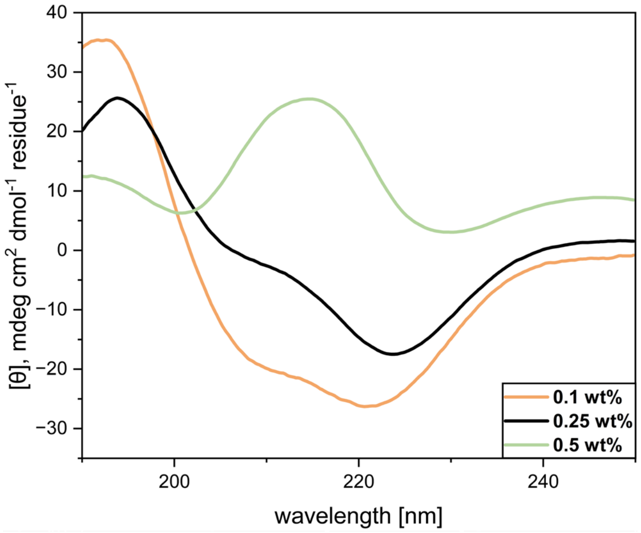

Table S9: hFF-K25H-GA-Gal

Abz LKKELAALKKELAALK**K**$_{(GA\text{-}Gal)}$ELAALK<u>**H**</u>EL

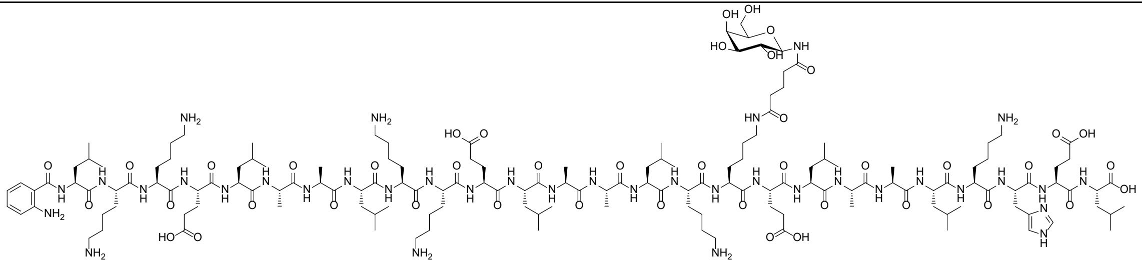

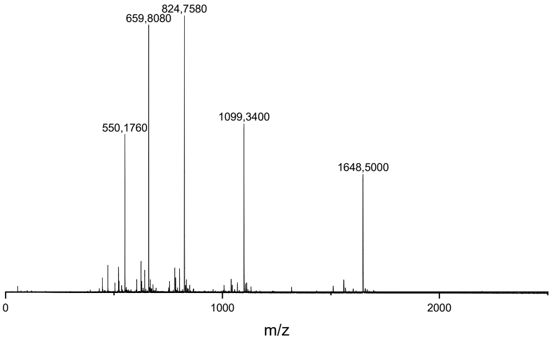

ESI-MS

	expected	received
$[M+2H]^{2+}$	1647.4687	1648.5000
$[M+3H]^{3+}$	1098.6458	1099.3400
$[M+4H]^{4+}$	824.2344	824.7580
$[M+5H]^{5+}$	659.5875	659.8080
$[M+6H]^{6+}$	549.8229	550.1760

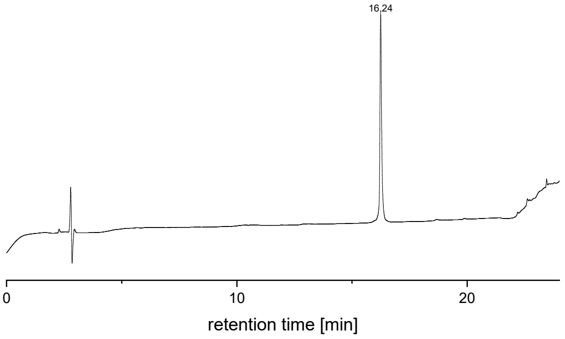

HPLC

10% ACN (0.1% TFA) to 60% ACN in Water (18 min)

retention time: 16.24 min

CD-concentration

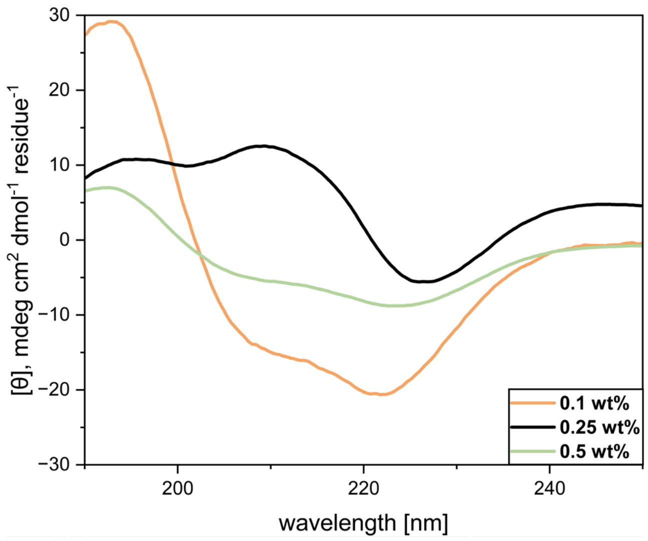

Table S10: hFF-K4H-K25H

Abz	LK**H**ELAALKKELAALKKELAALK**H**EL

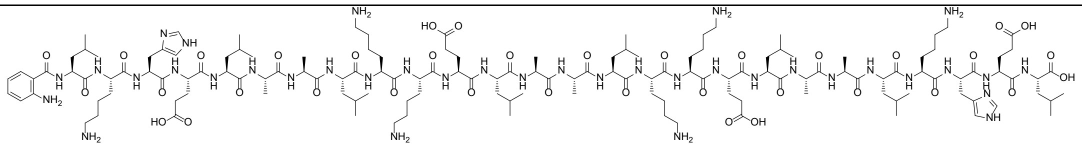

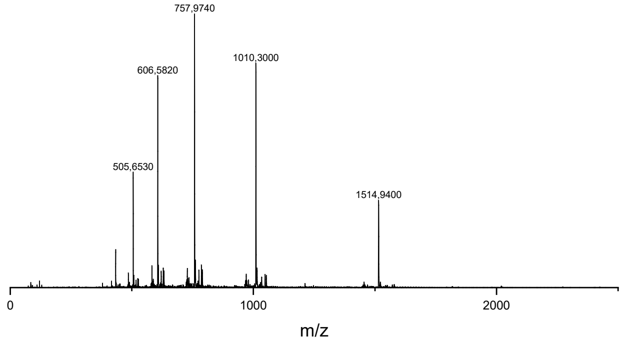

ESI-MS

	expected	received
$[M+2H]^{2+}$	1514.4004	1514.9400
$[M+3H]^{3+}$	1009.9336	1010.3000
$[M+4H]^{4+}$	757.7002	757.9740
$[M+5H]^{5+}$	606.3602	606.5820
$[M+6H]^{6+}$	505.4668	505.6530

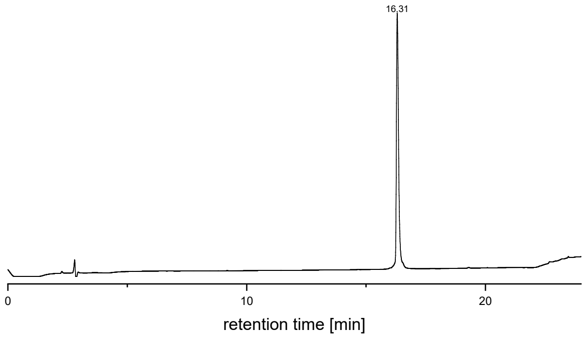

HPLC

20% ACN (0.1% TFA) to 60% ACN in Water (18 min)

retention time: 16.31 min

CD-concentration

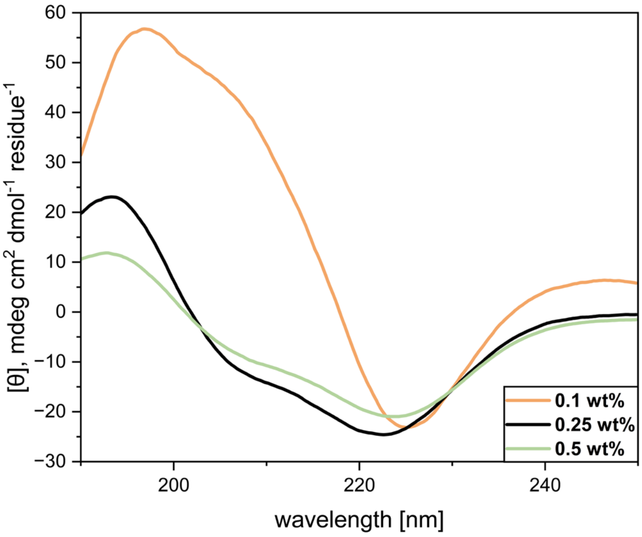

Table S11: hFF-K4H-K25H-GA-Man

Abz LK**H**ELAALKKELAALK**K**(GA-Man)ELAALK**H**EL

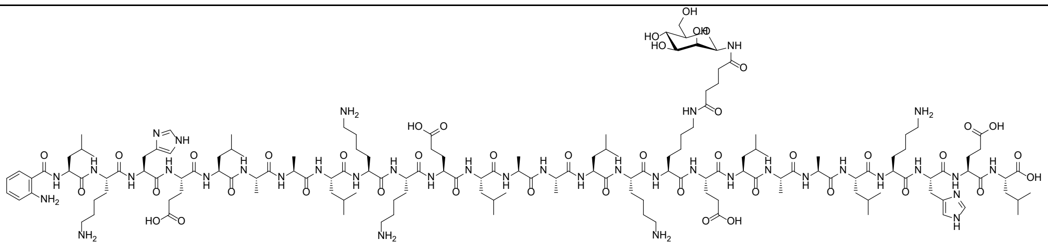

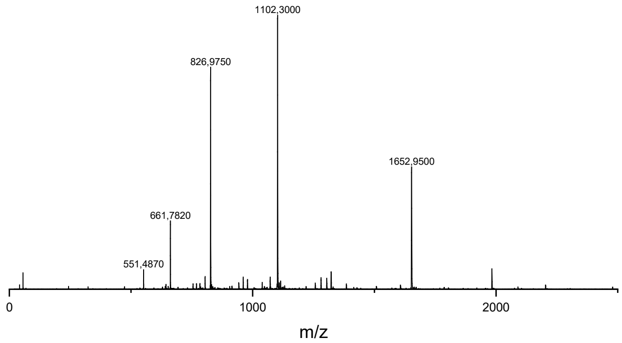

ESI-MS

	expected	received
$[M+2H]^{2+}$	1651.9506	1652.9500
$[M+3H]^{3+}$	1101.6338	1102.3000
$[M+4H]^{4+}$	826.4753	826.9750
$[M+5H]^{5+}$	661.3803	661.7820
$[M+6H]^{6+}$	551.3169	551.4870

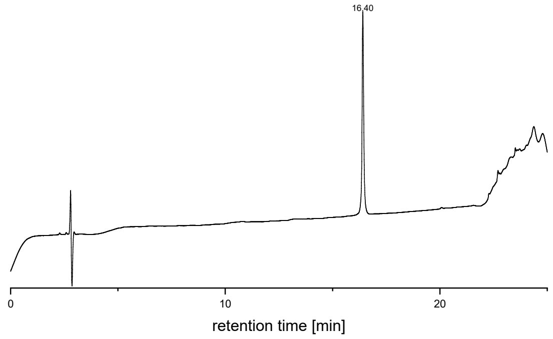

HPLC

10% ACN (0.1% TFA) to 60% ACN in Water (18 min)

retention time: 16.40 min

CD-concentration

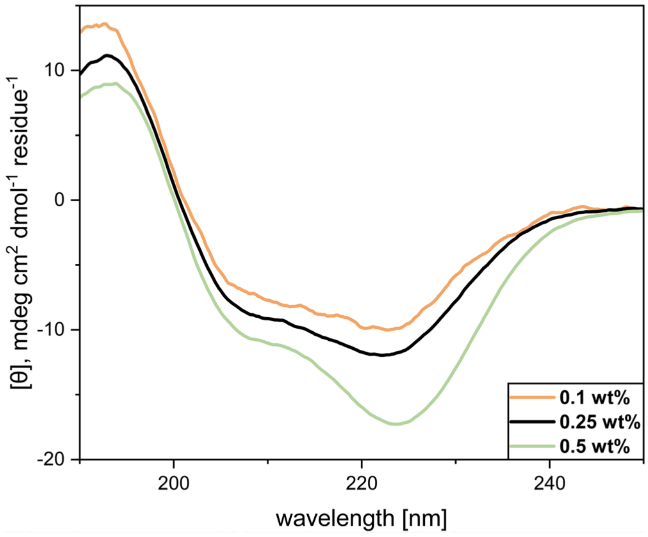

Table S12: hFF-K4H-K25H-GA-Gal

Abz LK**H**ELAALKKELAALK**K**(GA-Gal)ELAALK**H**EL

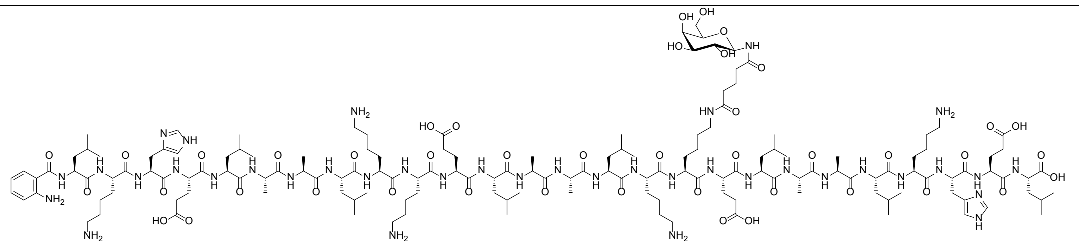

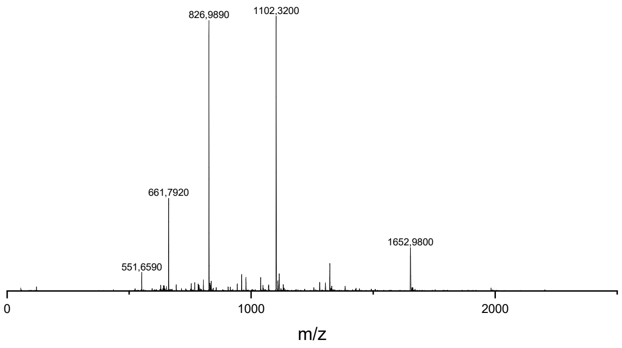

ESI-MS

	expected	received
$[M+2H]^{2+}$	1651.9506	1652.9800
$[M+3H]^{3+}$	1101.6338	1102.3200
$[M+4H]^{4+}$	826.4753	826.9890
$[M+5H]^{5+}$	661.3803	661.7920
$[M+6H]^{6+}$	551.3169	551.6590

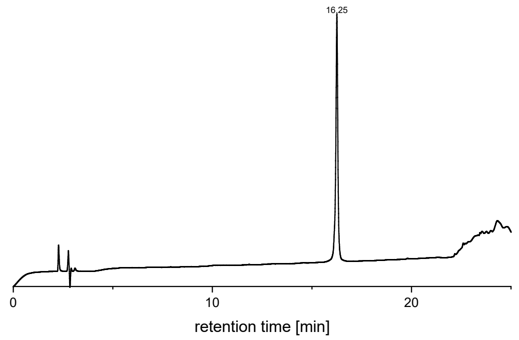

HPLC

10% ACN (0.1% TFA) to 60% ACN in Water (18 min)

retention time: 16.25 min

CD-concentration

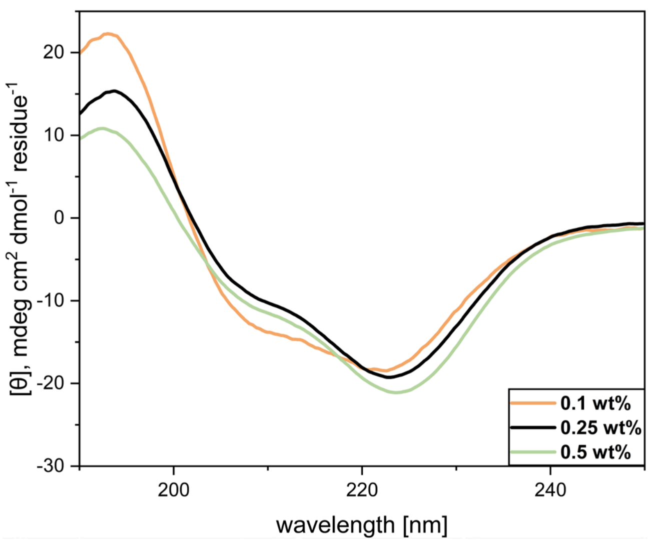

Circular Dichroism

Figure S1: hFF

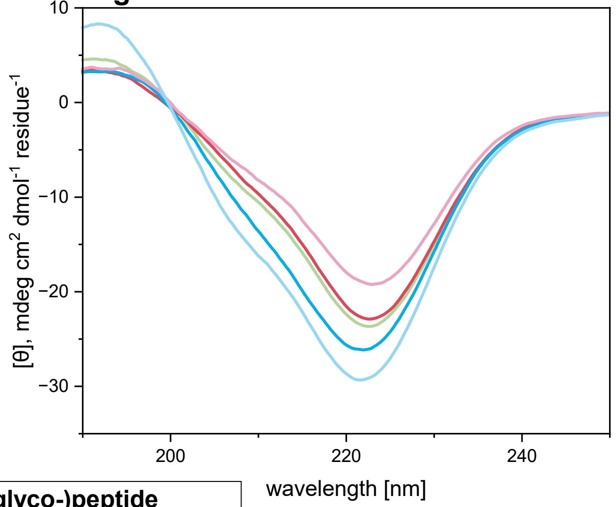

Figure S2: hFF-GA-Man

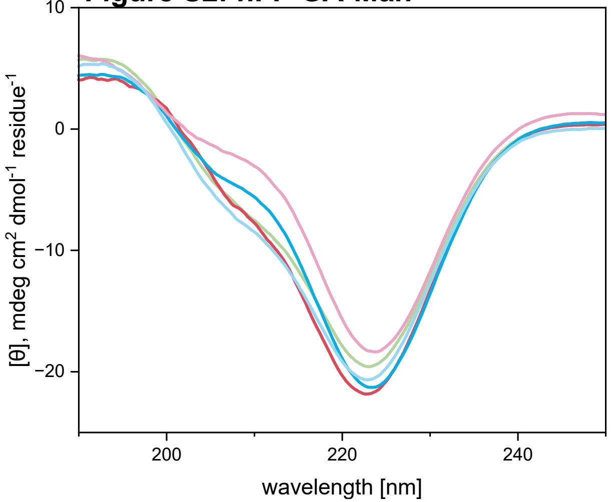

Figure S3: hFF-GA-Gal

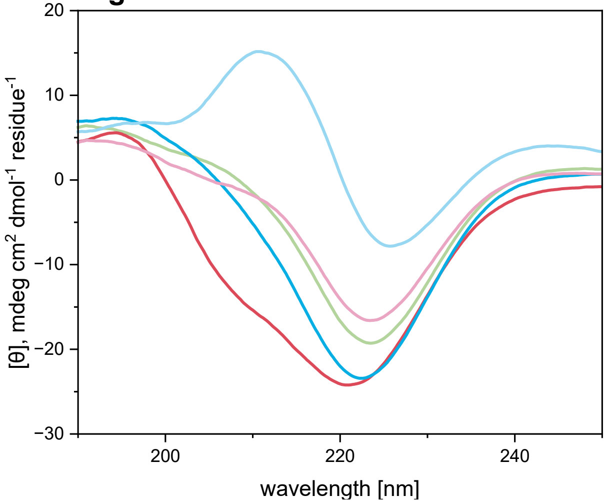

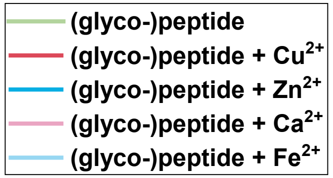

Figure S4: hFF-K4H

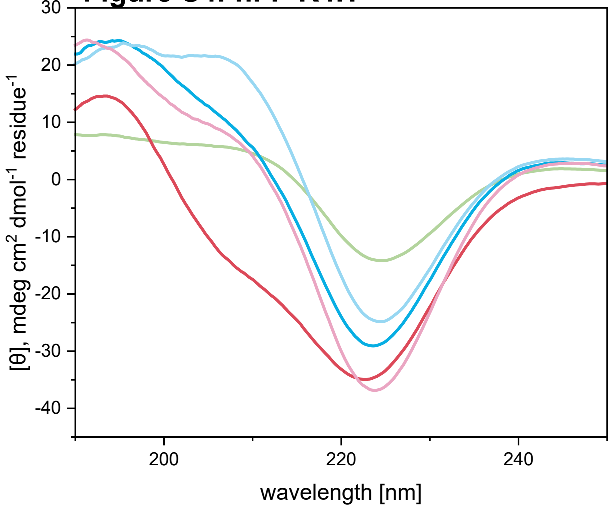

Figure S5: hFF-K4H-GA-Gal

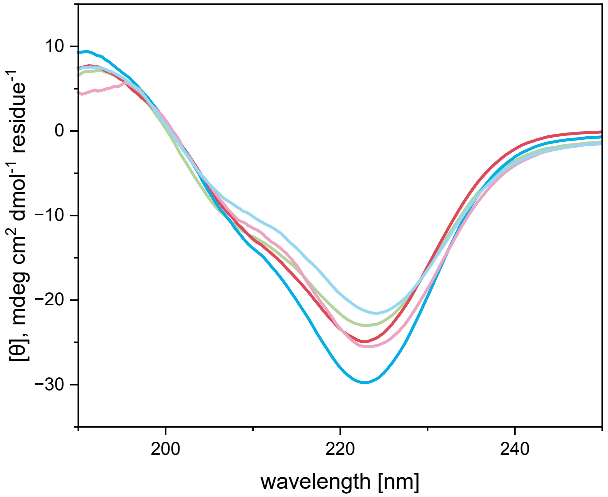

Circular Dichroism

Figure S6: hFF-K25H

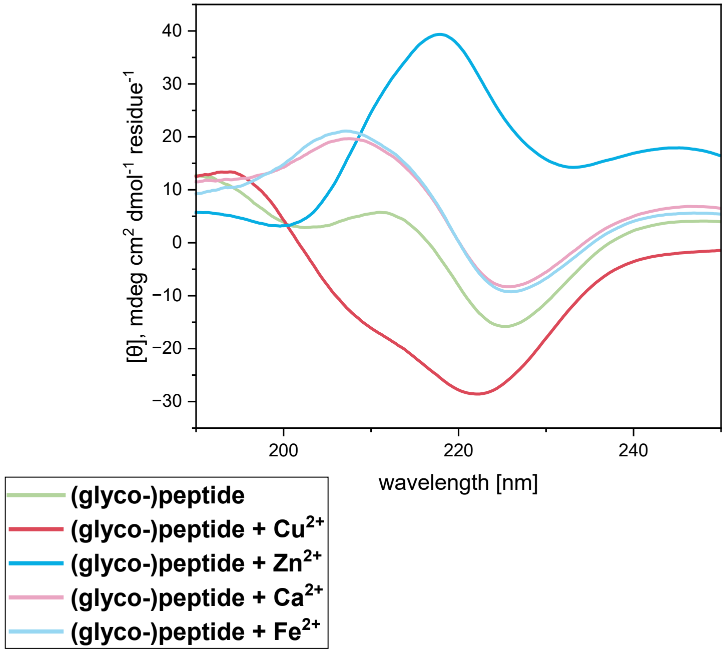

Figure S7: hFF-K25H-GA-Gal

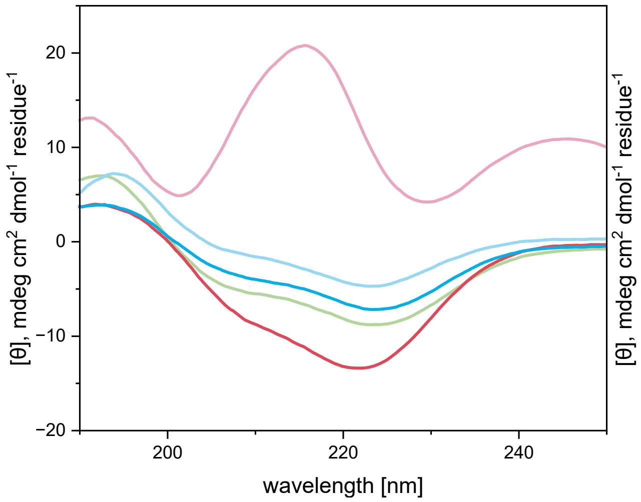

Figure S8: hFF-K4H-K25H

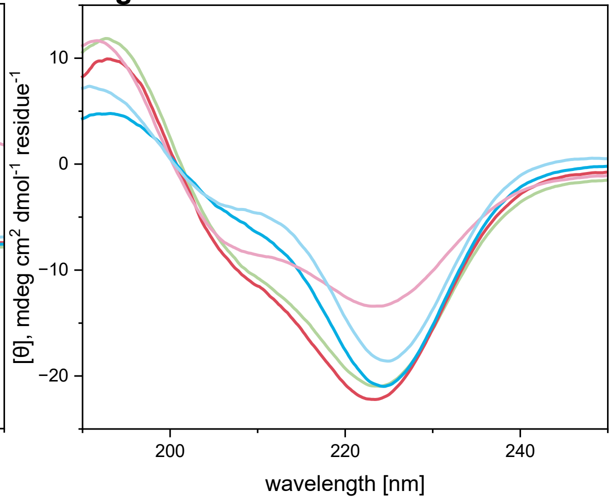

2. Rheology (S9 – S22)

Assessment of metal concentrations with copper

Previous experiments with different copper concentrations were performed on the undecorated peptides *hFF-K4H* and *hFF-K4H-K25H* to determine the concentration for the metal for the rheology and CD experiments.

Conditions: 1.65 mM, water pH 7.4, T = 37°C

Figure S9 - hFF-K4H + Cu^{2+} (1, 0.75, 0.5, 0.25 equiv.)

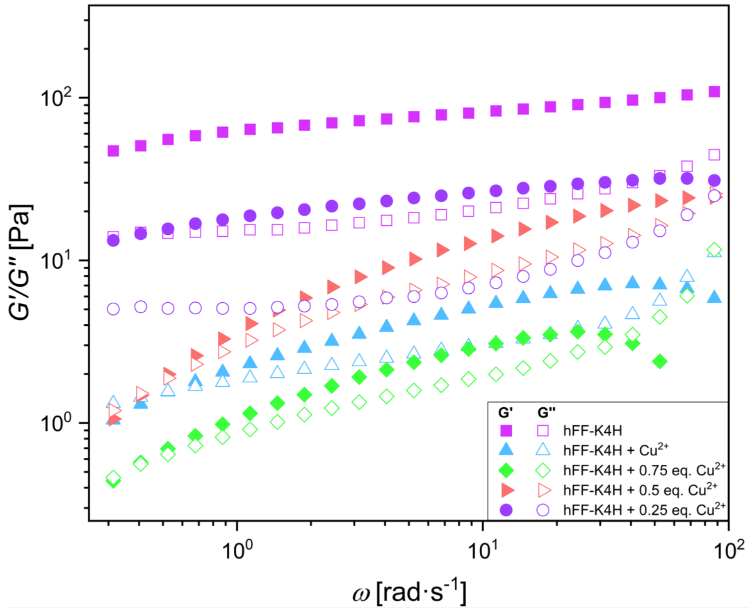

Figure S10 - hFF-K4H-K25H + Cu^{2+} (1, 0.75, 0.5 equiv.)

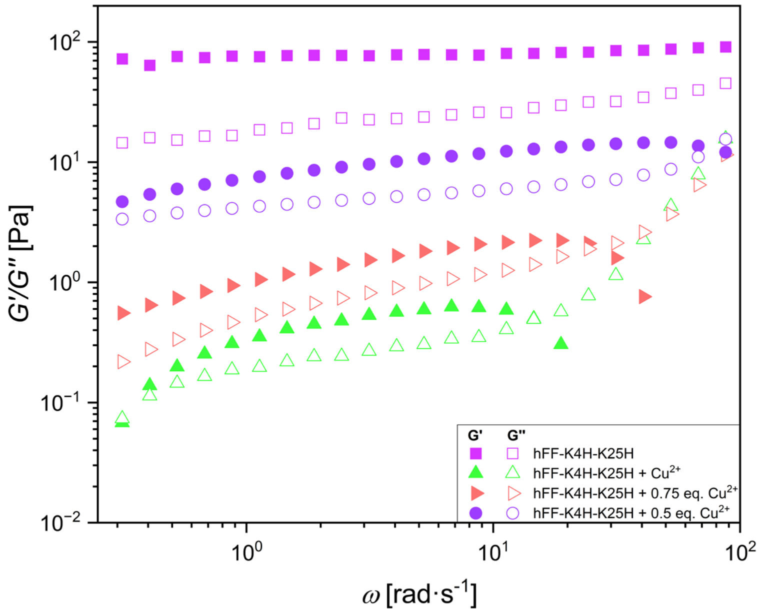

RHEOLOGY

Figure S11: hFF

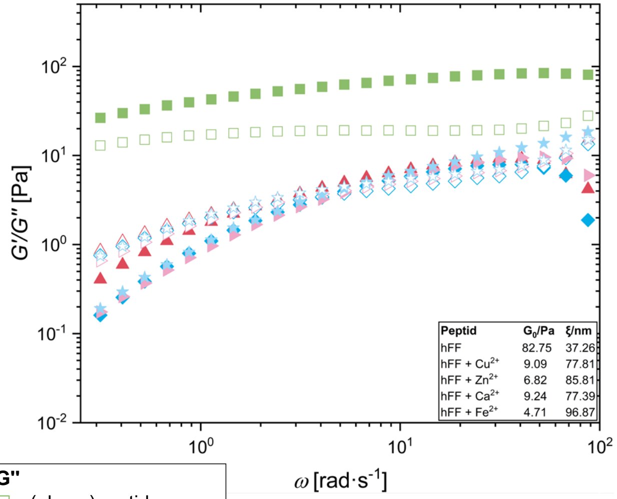

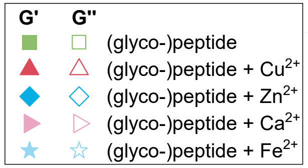

Figure S12: hFF-GA-Man

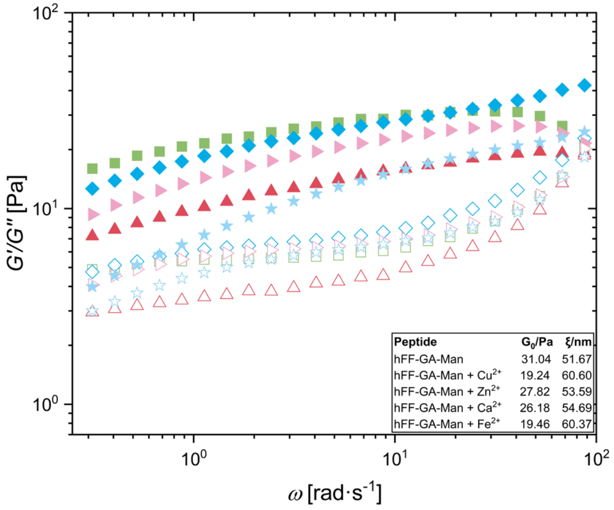

Figure S13: hFF-GA-Gal

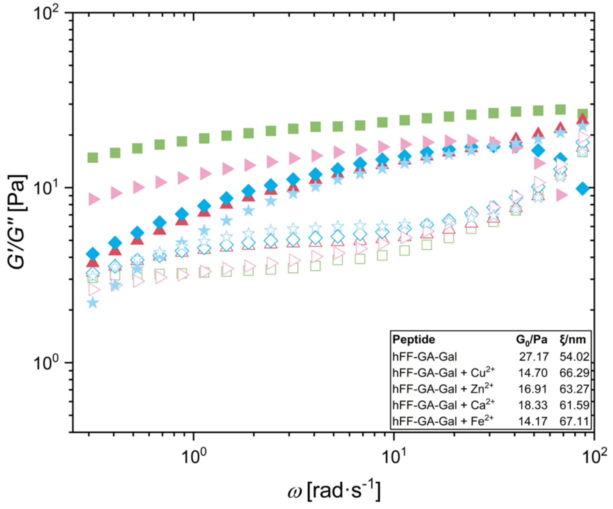

Figure S14: hFF-K4H

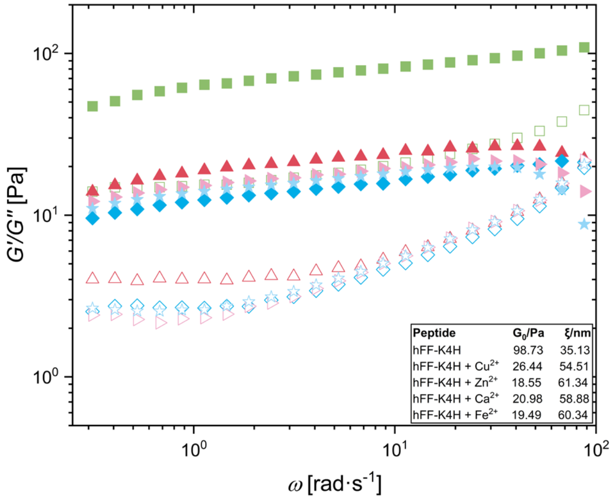

Figure S15: hFF-K4H-GA-Man

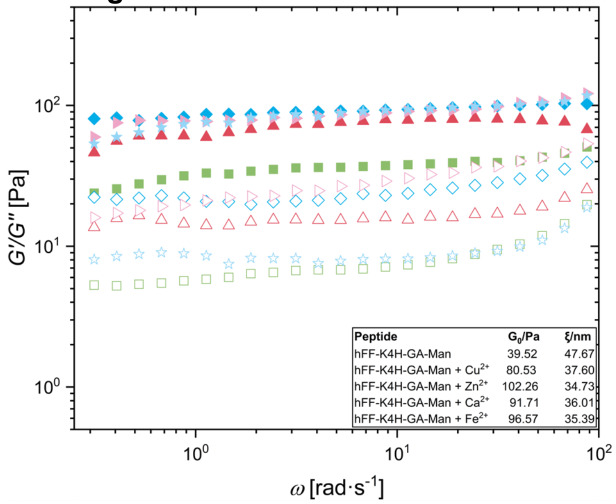

Figure S16: hFF-K4H-GA-Gal

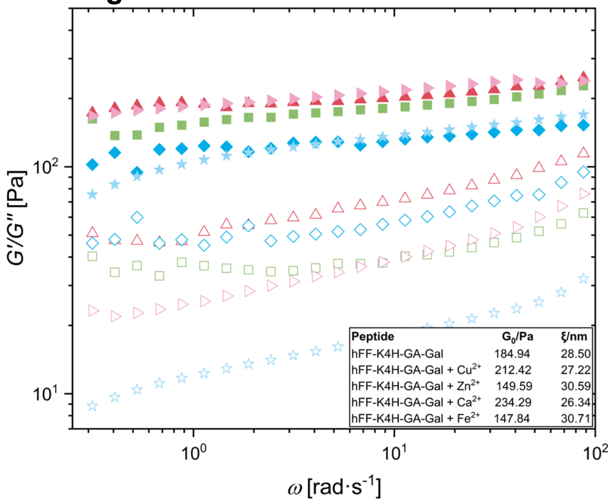

RHEOLOGY

Figure S17: hFF-K25H

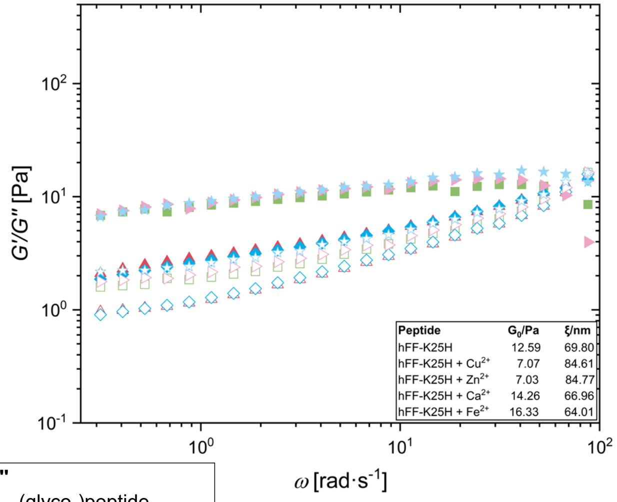

Figure S18: hFF-K25H-GA-Man

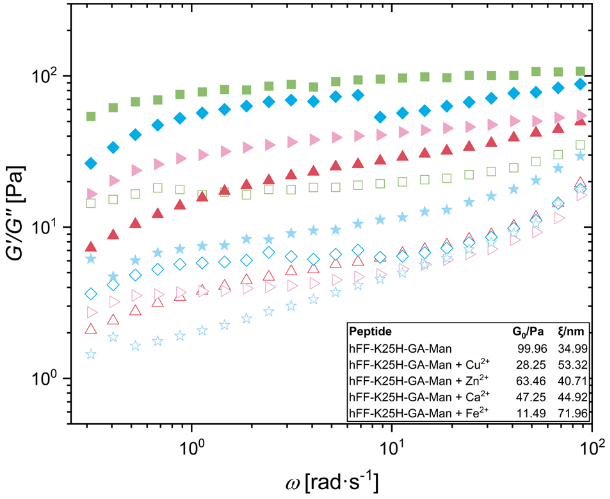

Figure S19: hFF-K25H-GA-Gal

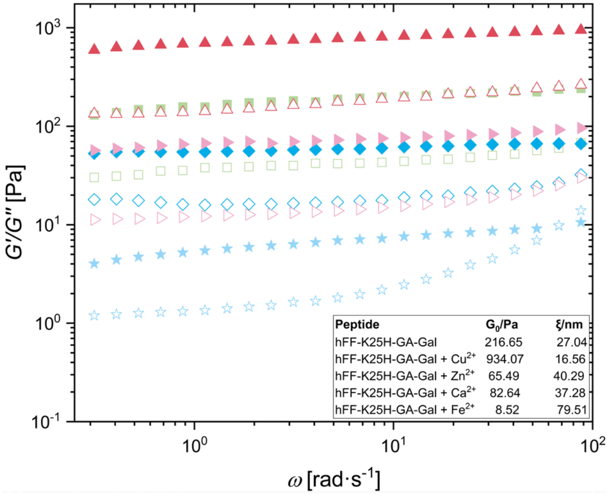

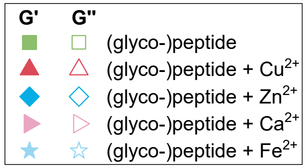

Figure S20: hFF-K4H-K25H

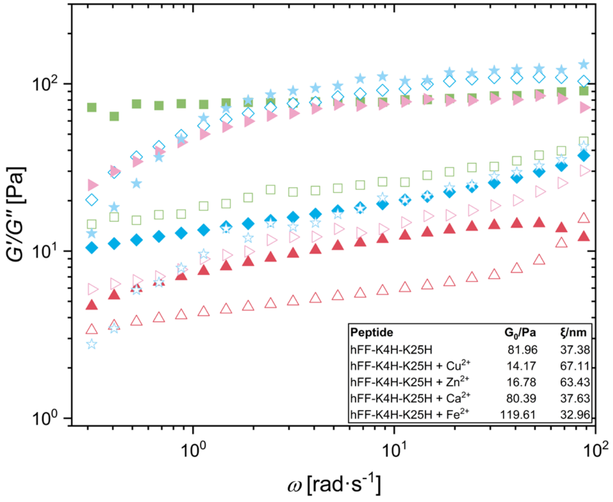

Figure S21: hFF-K4H-K25H-GA-Man

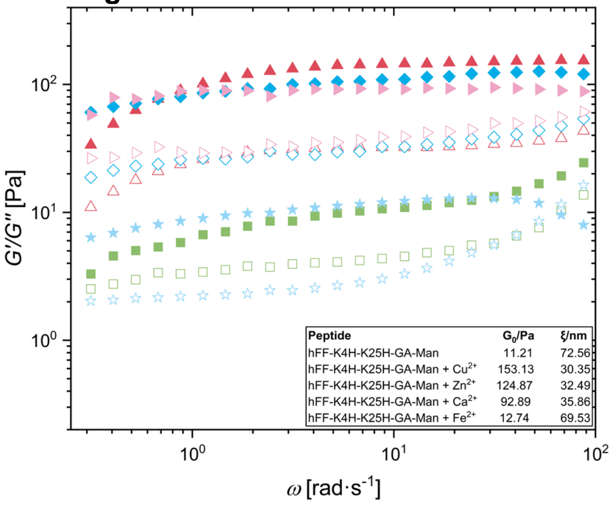

Figure S22: hFF-K4H-K25H-GA-Gal

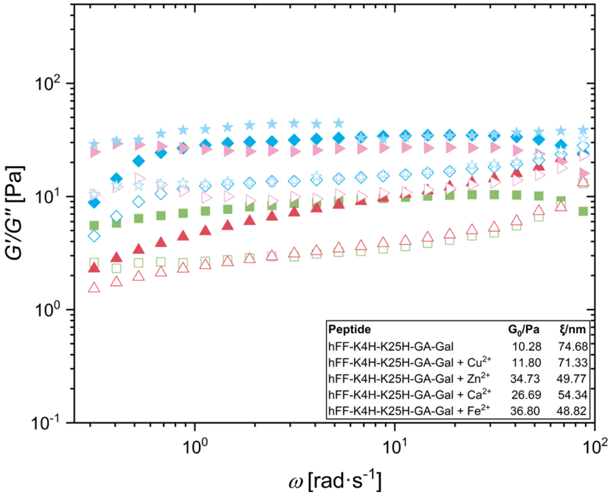

3. Transmission electron microscopy (TEM)

Figure S23: hFF-K4H-GA-Man

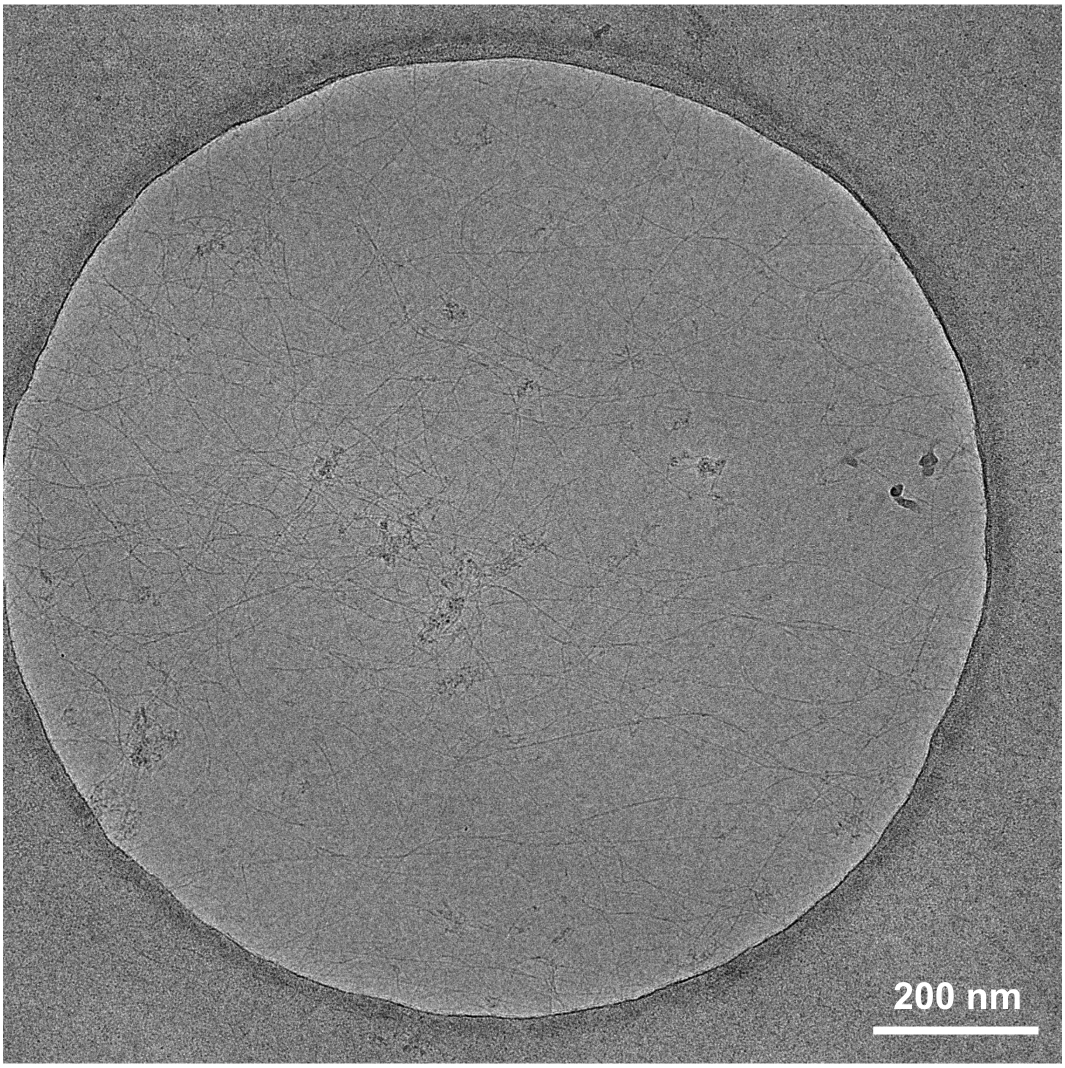

Transmission electron microscopy (TEM)

Figure S24: hFF-K4H-GA-Man + 0.25 equiv. Cu^{2+}

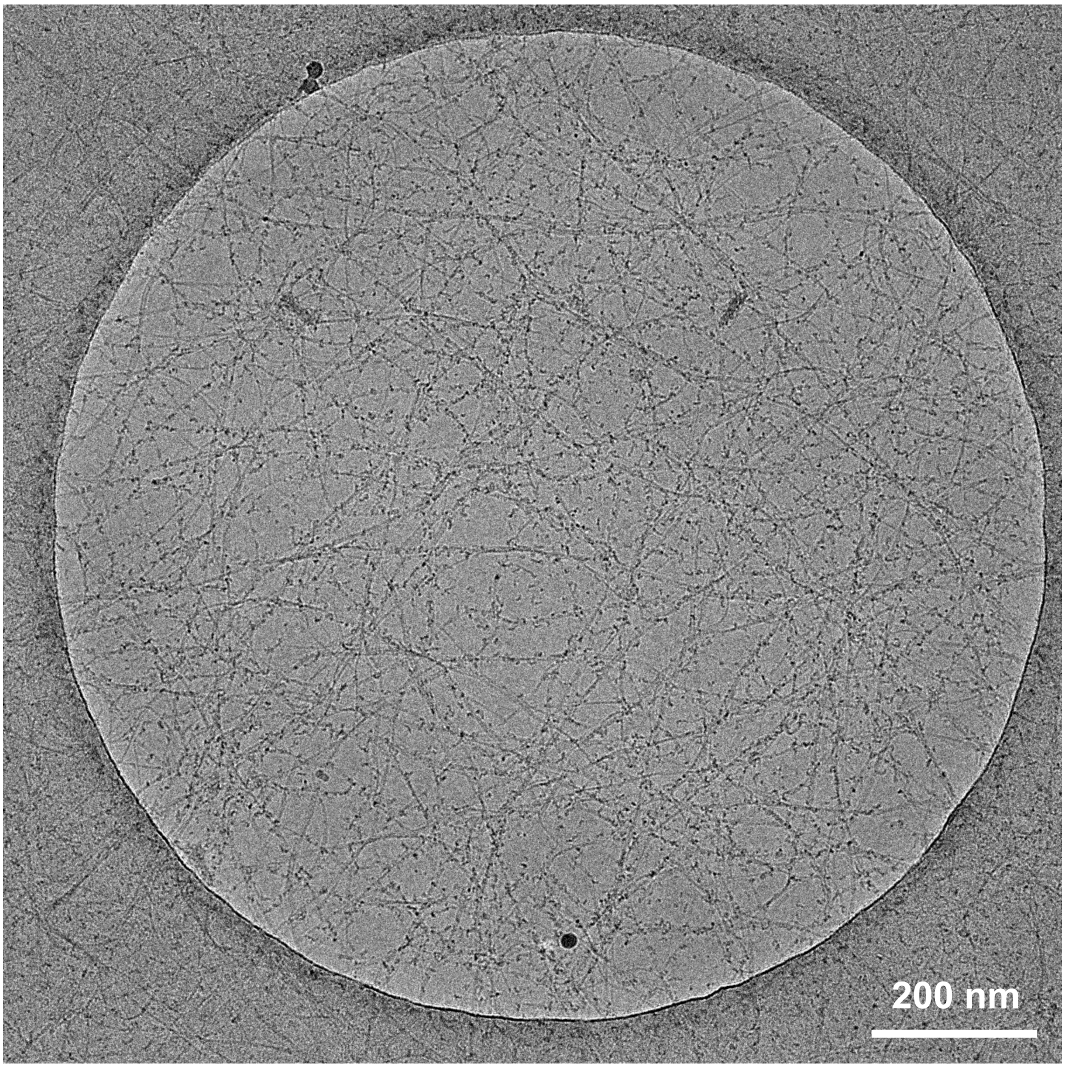

Transmission electron microscopy (TEM)

Figure S25: hFF-K4H-GA-Gal

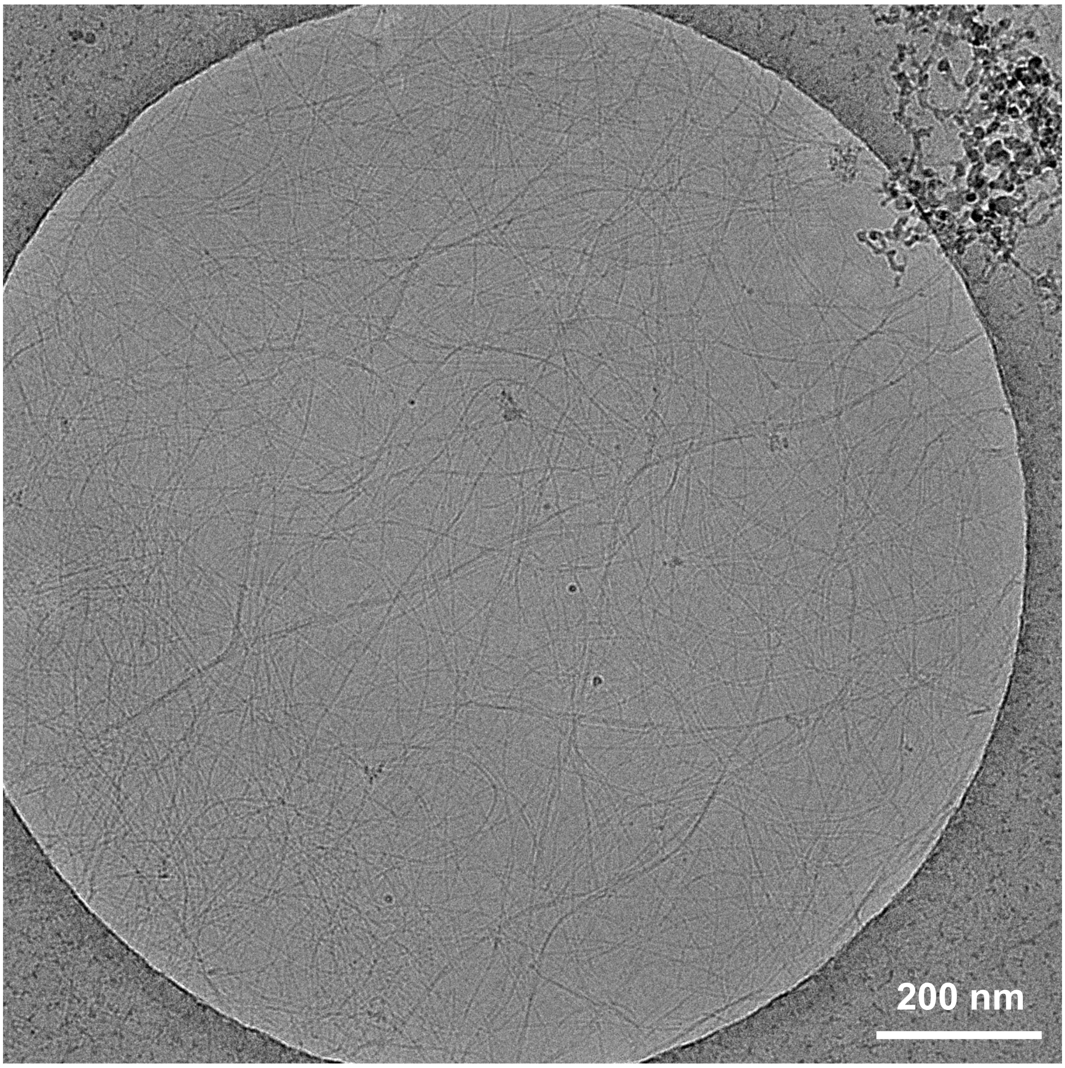

Transmission electron microscopy (TEM)

Figure S26: hFF-K4H-GA-Gal + 0.25 equiv. Cu^{2+}

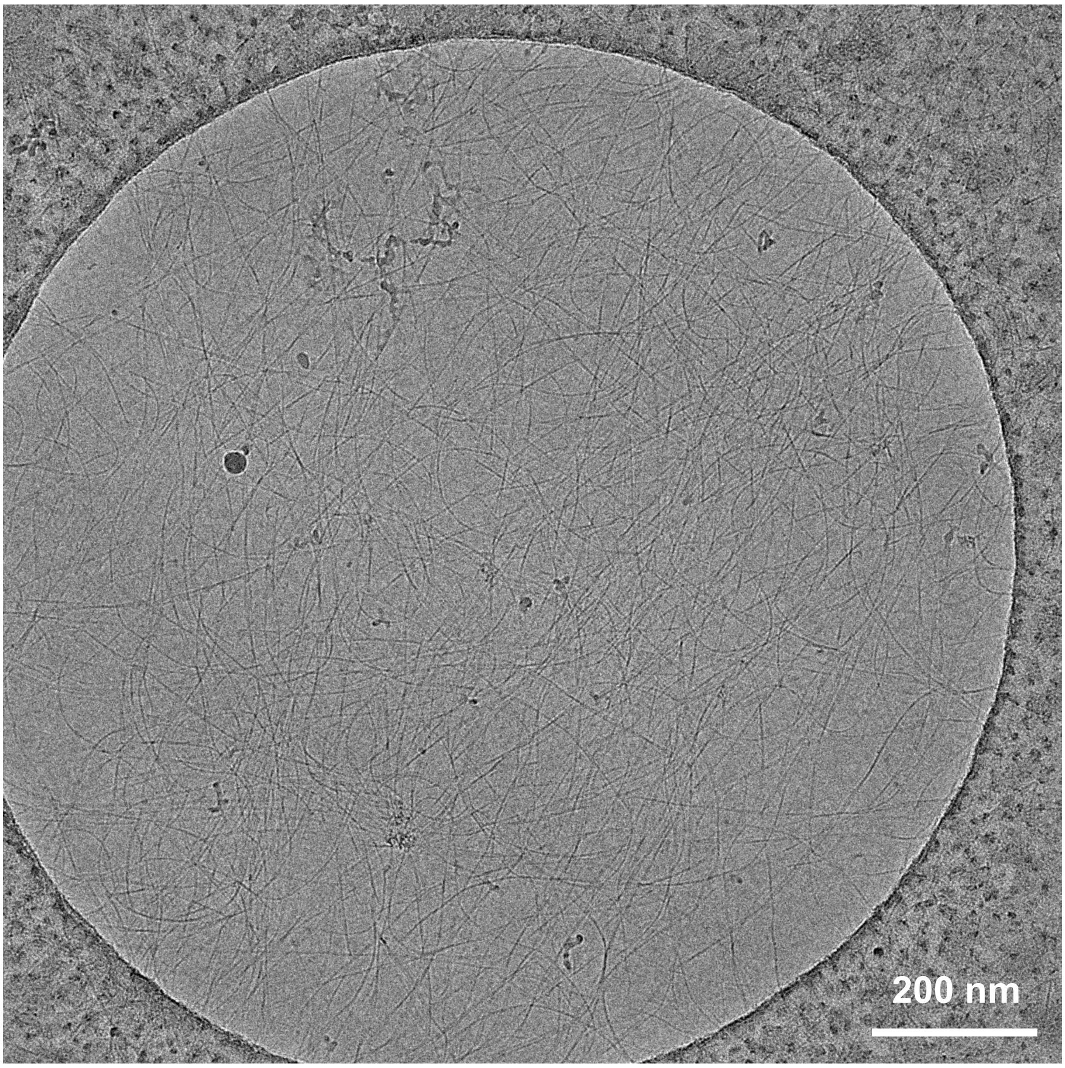

Transmission electron microscopy (TEM)

Figure S27: hFF-K25H-GA-Gal

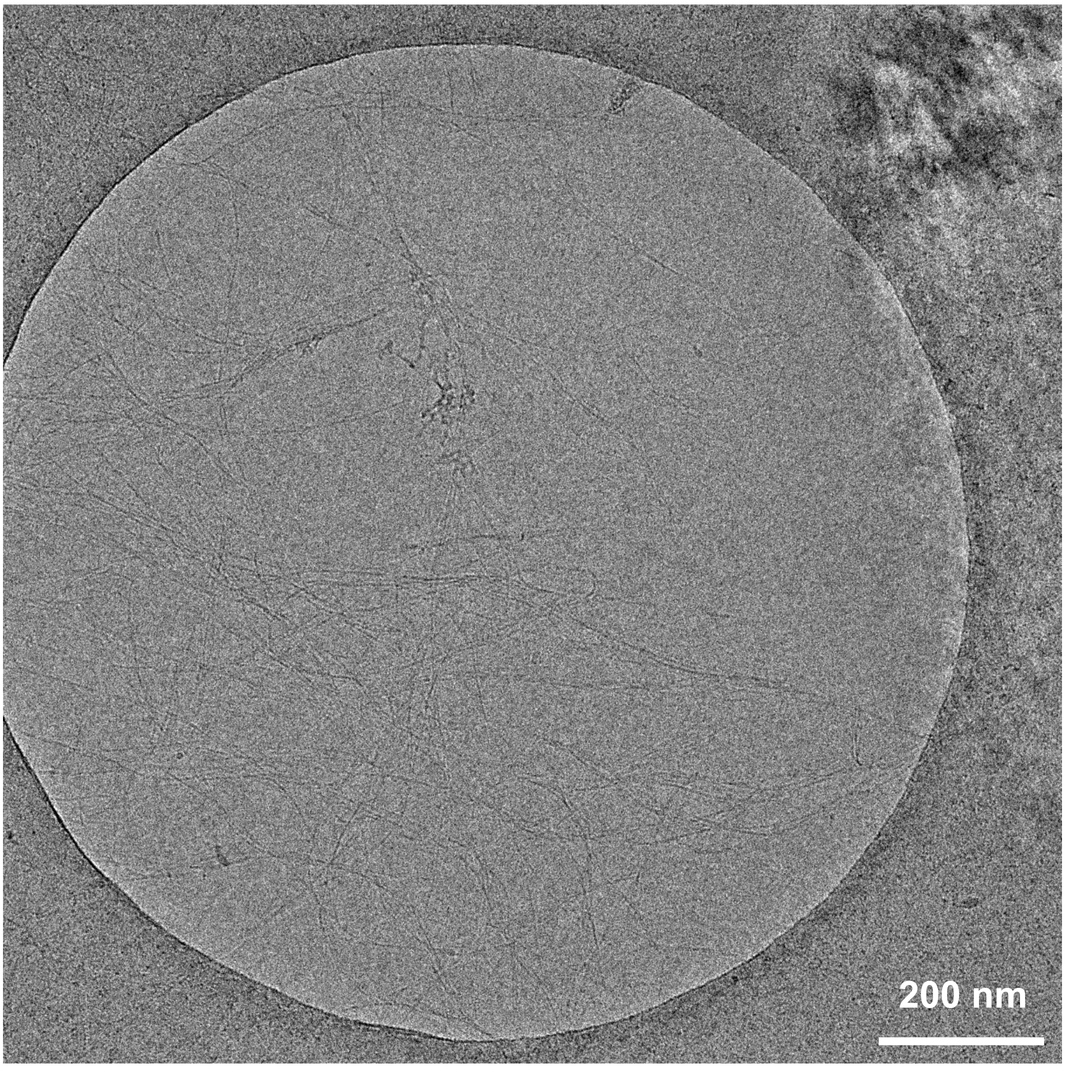

Transmission electron microscopy (TEM)

Figure S28: hFF-K25H-GA-Gal + 0.25 equiv. Cu^{2+}

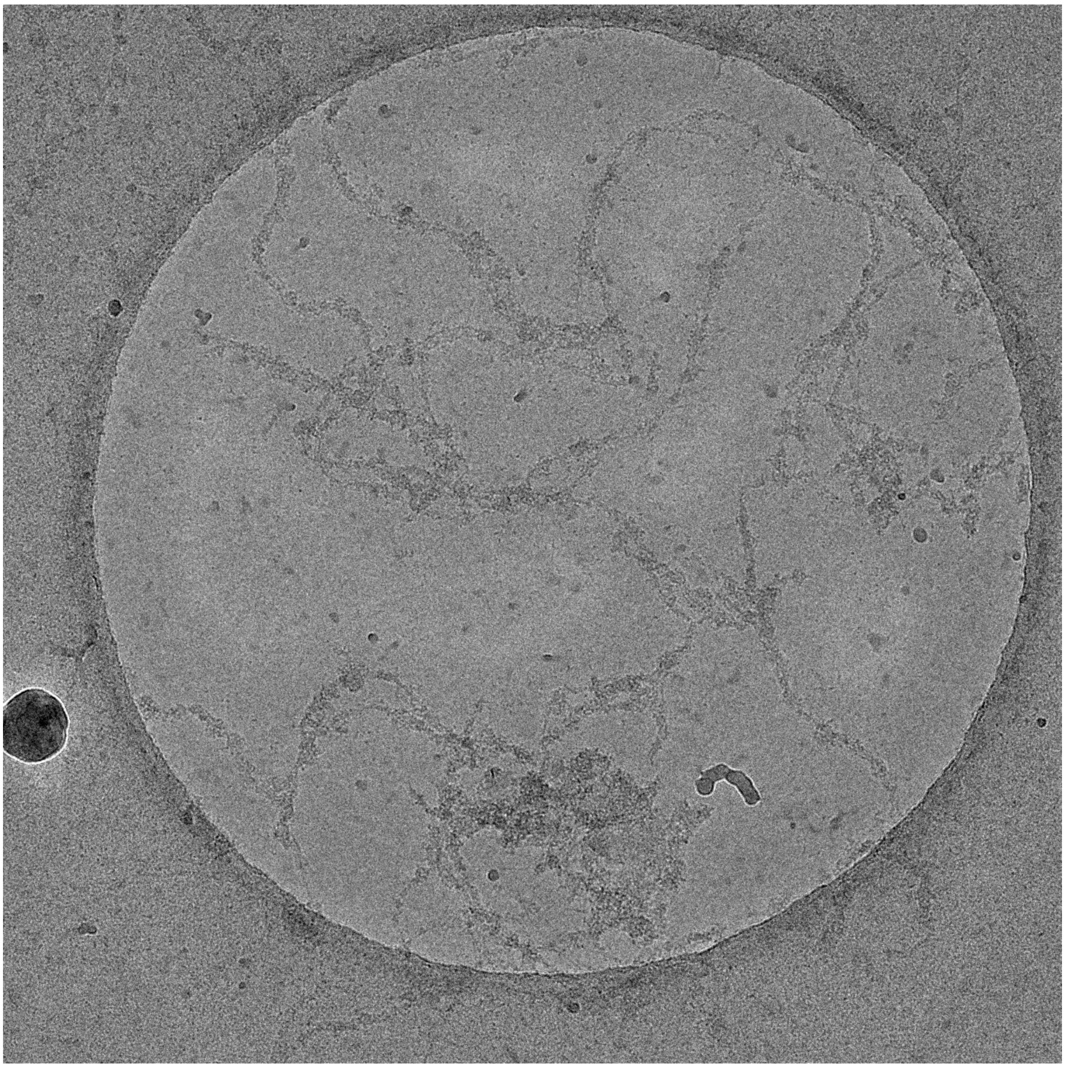

5. References

[1] Proksch, J.; Dal Colle, M. C. S.; Heinz, F.; Schmidt, R. F.; Gottwald, J.; Delbianco, M.; Keller, B. G.; Gradzielski, M.; Alexiev, U.; Koksch, B. Impact of Glycan Nature on Structure and Viscoelastic Properties of Glycopeptide Hydrogels. **2024**. https://doi.org/10.1002/psc.3599.
